# Goat temporomandibular joint osteochondral defects for future regenerative studies of osteoarthritis-like degeneration

**DOI:** 10.1371/journal.pone.0356421

**Published:** 2026-08-19

**Authors:** Mairobys Socorro, Xudong Dong, Juan M. Taboas, Alejandro J. Almarza

**Affiliations:** 1 Department of Oral and Craniofacial Sciences, Center for Craniofacial Regeneration, University of Pittsburgh School of Dental Medicine, Pittsburgh, Pennsylvania, United States of America; 2 Department of Bioengineering, Swanson School of Engineering, University of Pittsburgh, Pittsburgh, Pennsylvania, United States of America; Ohio State University, UNITED STATES OF AMERICA

## Abstract

Temporomandibular joint osteoarthritis (TMJ-OA) is a type of temporomandibular disorder (TMD) characterized by structural changes in the joint due to the gradual degeneration and loss of articular cartilage, as well as remodeling of the underlying bone. The complexity of these structural and functional changes underscores the importance of evaluating regenerative therapies in large- animal models that closely mimic human joint physiology prior to clinical translation. Considering that TMJ cartilage consists of fibrocartilage, dissimilar from hyaline cartilage found in other diarthrodial joints, and that osteochondral defects (OCDs) are reproducible injuries for developing regenerative therapies for osteoarthritis (OA), this study aimed to establish a suitable large-animal model of OCD in the TMJ condyle of goats. We surgically generated and compared two types of OCDs, a novel tunnel defect drilled retrograde from the posterior aspect of the condyle to the articular surface and a superior defect drilled anterograde. Live computed tomography (CT) and histological assessment showed that the defects were critically sized, with incomplete subchondral bone healing and no cartilage regeneration up to 60 days post-surgery. All defects were filled with fibrous tissue throughout and woven bone at the periphery. The articular fibrocartilage surrounding both defect types displayed signs of arthritic degeneration per the modified OARSI scale, including the loss of the zonal structure (fibrous, transition, and cartilaginous layers). Given similar impaired healing, the tunnel OCD is favorable to preserve the disc attachments and minimize surgical manipulation of the TMJ while creating larger defects and OA-like degeneration in the goat. In summary, this study presents a practical preclinical goat model for evaluating future regenerative therapies targeting TMJ-OA.

## Introduction

Temporomandibular disorders (TMDs) refer to a group of conditions affecting not only the temporomandibular joint (TMJ) but also the surrounding masticatory muscles and adjacent structures [1–3]. TMDs have a multifactorial etiology, which includes injury, arthritis, muscle tension due to stress, genetic factors, aging, dislocation, and structural issues [4,5]. It is estimated that as high as 31% of the adult population is affected by TMDs [6], and that over 10 million US adults develop clinically verified TMDs every year [7]. There are approximately 30 different types of TMDs that result in chronic pain and significantly affect the patient’s quality of life [8]. Besides the joint pain experienced by TMD patients, other signs and symptoms of TMD include headaches, limited mouth opening, difficulty chewing and swallowing food, deviation, clicking, dislocation, and even locking of the jaw in severe cases [9–11].

TMJ osteoarthritis (TMJ-OA) is a type of TMD, characterized by the progressive degeneration of cartilage and damage to the underlying subchondral bone in the TMJ condyle [2]. Although TMJ-OA often involves degenerative changes in the condyle, articular disc, and mandibular fossa, many studies have reported that the condyle is the most severely affected site [12–14]. The degeneration of the condylar surface may contribute to the pain, structural changes, and dysfunction observed in individuals with TMJ-OA. As such, condylar regeneration is a practical, clinically relevant focus for developing early-stage therapeutic strategies in TMJ-OA. Despite advances in the field, there are currently no regenerative treatment options for TMJ-OA, and further investigation is needed to develop effective therapies [14].

Preclinical models of TMJ‑OA are essential for understanding disease mechanisms and evaluating potential regenerative therapies. A wide variety of TMJ‑OA preclinical models have been developed and broadly classified into induced models (including chemical, surgical, and mechanical approaches); naturally occurring models (including genetically modified species). Chemically induced models, such as intra-articular injection of chemical agents (e.g., monosodium iodoacetate, collagenase, or complete Freund’s adjuvant) can produce rapid and reproducible cartilage degeneration and subchondral bone changes, with ease of induction and adjustable severity. However, they primarily mimic inflammatory or cytotoxic pathways rather than structural injury per se. Surgical and mechanical models, including displacement, partial discectomy, and perforation of the disc, aberrant bite occlusion, and impact/trauma protocols, induce osteoarthritis through joint instability and biomechanical alterations. Yet, they often require complex procedures, extended time courses for disease progression, and variable pathology [15,16].

In contrast, focal cartilage and osteochondral defects (OCDs) enable the creation of well-defined, reproducible lesions, making them particularly valuable for studies aimed at repairing the osteochondral interface [17]. These defects facilitate evaluation of how regenerative constructs integrate with host tissue, the attachment and stability of engineered cartilage or bone grafts, and the effects of implants on both cartilage repair and subchondral bone remodeling.

Most preclinical models of TMJ-OA rely on small animal models, primarily mice, rats, and rabbits, due to their cost-effectiveness, genetic tractability, and suitability for mechanistic studies; however, these models have limited capacity to replicate the biomechanical and anatomical complexity of the human TMJ [18]. While small animal models have been valuable for proof-of-concept studies, their smaller joint size and biomechanical differences may not accurately recapitulate treatment efficacy observed in human TMJ-OA, thereby limiting the translational evaluation of implantable regenerative materials [15]. On the other hand, larger animal models offer joint dimensions, cartilage thicknesses, and biomechanical loads that more closely approximate the human TMJ. Additionally, they provide clinically relevant defects of similar size, biomechanics, and physiology. Given the critical role of large-animal models in validating the biomechanical and anatomical efficacy of emerging regenerative therapies, there remains a need for an innovative large-animal model that facilitates the development of interventions for TMJ-OA.

The porcine OCD model can replicate OA-like changes [19], but it has yet to be applied for evaluating implant-based cartilage regeneration strategies in the TMJ. Most cartilage regeneration studies using the mini-pig model have focused on the knee joint, particularly the medial and femoral condyles, as well as the femoral trochlea. Though pigs have a TMJ size closer to humans than small animals, the joint is surgically less accessible than in humans and other species like goats or sheep [20], which limits implant placement and handling of human-scale biomaterials. Consequently, species with larger, more surgically accessible TMJs are better suited for evaluating implant-based regenerative strategies, thereby guiding the selection of goats over other large-animal models.

Although goats and sheep are comparable models, goats are more easily procured at skeletally mature ages from local farms, making them a more practical option. In support of this, Li et al. [21] demonstrated that different types of trauma (disc displacement, cartilage defect, and the combination of both) produce distinct degenerative profiles in the goat TMJ. Importantly, the combined injury resulted in the most severe and rapid OA changes, highlighting the capacity of the caprine model to reproduce clinically meaningful disease progression. The goat model also provides a rigorous testing environment for therapeutics. As ruminants, goats chew their food for 12–18 hours each day, increasing cyclic loading by an order of magnitude compared to humans and thereby accelerating durability and wear testing. Thus, goat models have come into favor for TMJ OCDs, which commonly involve a surgically induced superior approach [18].

Several goat OCD models have been established to study TMJ cartilage and osteochondral regeneration. Zhu et al. [22] created 3 mm × 5 mm OCDs (superior defect) in 25- to 28-month-old male Eastern cross goats, providing a valuable approach for evaluating tissue repair. On the other hand, Sun et al. [23] induced full-thickness condylar cartilage defects in younger goats (6–8 months old, 10–22 kg), enabling assessment of cartilage-specific regenerative strategies. More recently, Yu et al. [24] generated 5 mm × 5 mm OCDs (superior defect) in 12-month-old goats, demonstrating early cartilage repair with promising histological outcomes. Despite their advances, these models have notable limitations, including undisclosed mechanical function, reliance on relatively young animals with greater regenerative capacity, and restriction to male goats—an important shortcoming given the higher prevalence of TMJ-OA in females. These gaps underscore the need for a standardized, translationally relevant goat model to evaluate implant-based regenerative therapies for TMJ OCDs.

Herein, this study aimed to establish a suitable large-animal model of OCD in the TMJ condyle of goats. We surgically generated and compared two types of OCDs: a novel tunnel defect drilled retrograde from the posterior aspect of the condyle to the articular surface and a superior defect drilled anterograde. We monitored bone regeneration in the OCDs over time using in vivo CTs and assessed healing of the untreated OCDs and OA-like degeneration of the surrounding condylar cartilage with histochemical analysis at 60 days post-operation. Unoperated condyles from age-matched animals were used as anatomical and histopathological controls.

## Materials and methods

### Animals

Three- to four-year-old healthy Boer female goats (n = 10) were used to surgically generate OCDs (superior or tunnel) in the TMJ condyle. Additionally, three age-matched goats served as native controls (n = 3). These goats were purchased from May Farms (PA) and were approximately 47.3 ± 6.9 Kg at the time of the surgery. All animal procedures were performed in strict accordance with the recommendations in the Guide for the Care and Use of Laboratory Animals of the National Institutes of Health. The protocol was approved by the University of Pittsburgh Institutional Animal Care and Use Committee (IACUC Protocol # 23083590). Animals were euthanized by intravenous administration of an approved euthanasia solution (Euthasol: pentobarbital sodium and phenytoin sodium) 60 days after condyle surgery. Necropsied tissues were then processed for histological evaluation.

### Surgical procedures to create osteochondral defects (OCDs) in the goat TMJ condyles

Using standard operating procedures to ensure consistency and efficiency of the model, OCDs were surgically created in the TMJ condyles of goats under general anesthesia with endotracheal intubation and placed in a lateral decubitus position. Before surgery, goats were fasted for 24 hours. On the day of surgery, the goats were sedated using ketamine (5 mg/kg) administered by intravenous injection, followed by induction with propofol (4–6 mg/kg). Isoflurane (2–3%) was administered by inhalation throughout the entire surgery. The surgical area was shaved and disinfected using povidone-iodine and ethanol. Then, Lidocaine (2%) was subcutaneously injected around the surgical opening. Surgical access to the TMJ was achieved by making an incision near the temporal lobe of the goat, followed by careful separation of the surrounding musculature to expose the joint space. During the surgical procedure, the surrounding tissues were carefully separated using specialized surgical retractors. These instruments provided clear visibility and access to the operative site while minimizing trauma to the adjacent muscles, fascia, and neurovascular structures. After the joint space was identified, the OCDs (tunnel or superior defects) were created as described in the following section. Following surgery, the muscle, fascia, and skin were closed in layers with resorbable sutures using standard surgical techniques.

### Generation of OCDs in the TMJ condyle

Two different types of OCDs were surgically created in the TMJ condyle of skeletally mature female goats (n = 10), an oblique superior OCD by anterograde drilling (n = 4), similar to prior OCD models [25–27], and a novel tunnel OCD by retrograde drilling (n = 6). Both OCD types were left empty and were allowed to heal for up to 60 days. The biological replicates were evenly distributed between the left and right TMJs. Using the contralateral joint as a control could introduce confounding variables due to compensatory loading, altered biomechanics, and systemic responses that may affect both sides. Therefore, unoperated animals served as controls (n = 3).

#### Superior OCD.

**Fig 1:**
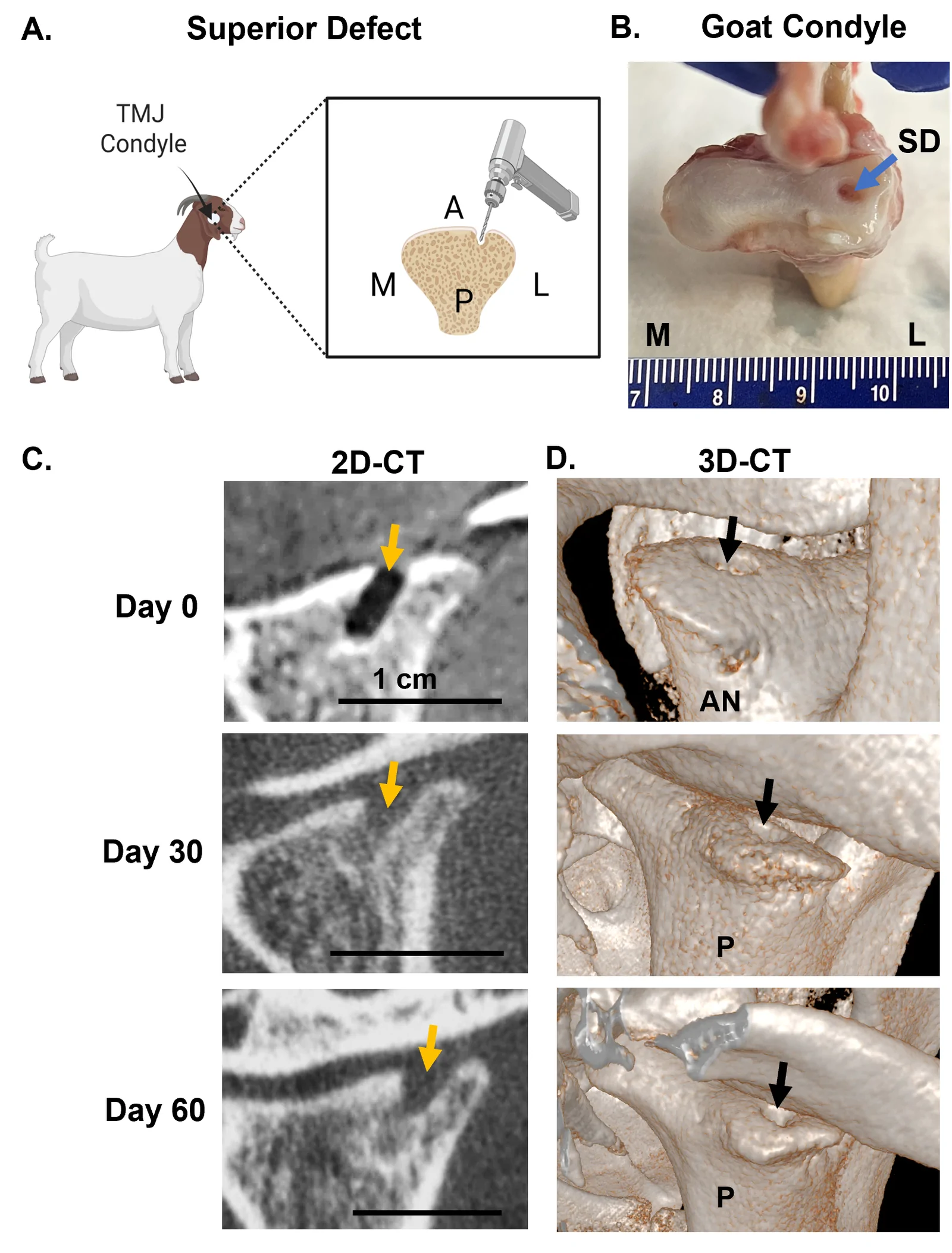
Superior defect in a goat TMJ condyle monitored by CT imaging and gross morphology. (A) Schematic representation of the superior defect (SD) surgically generated in the TMJ condyle of adult female goats. The diagram shows the alignment and positioning of the drill bit to generate superior defects. Key anatomical landmarks are labeled to provide a reference for the defect’s location. (B) Representative digital photograph showing the gross morphology of the TMJ condyle 60 days after the creation of the superior OCD. The blue arrow indicates the SD. (C-D) Representative two-dimensional (2D) images (coronal view) and three-dimensional (3D) reconstruction of CT scans illustrating the location and incomplete healing of the SD up to 60 days post-surgery without intervention. Yellow arrows indicate the defect location in the 2D images at 0, 30, and 60 days, while black arrows highlight the OCDs on the condyle surface in the 3D CT images. Scale bars are 1 cm. Medial (M), lateral (L), anterior (AN), posterior (P).

A 3 cm anteroposterior cutaneous incision was made over the TMJ, and the overlying muscle was separated using blunt dissection. The TMJ disc was liberated by cutting lateral attachments in the anterior and posterior aspects of the disc. This allowed one-half of the disc to be folded over itself to access the condylar surface. Next, a flat (0.5 mm thick), rounded stainless steel spatula was used to lift the disc and avoid damage. An oblique OCD measuring 2.4 mm in diameter was created using a surgical drill (Conmed Hall PRO6200 Mpower) and a 2.4 mm bone drill bit (Medtronic) from the superior-lateral aspect to the inferior-medial aspect of the TMJ condyle (Fig 1A). The drill was positioned on the condylar surface and advanced to a maximum depth of 5 mm below the surface of the mandibular condyle. After making the superior OCD, natural blood clot formation in the OCD was allowed before suturing the muscle and skin. The use of a 2.4 mm drill bit enabled penetration of the condylar surface at an angle greater than 45°. In comparison, a larger drill bit would result in more acute angles of entry due to shank interference with the TMJ disc.

#### Tunnel OCD.

**Fig 2:**
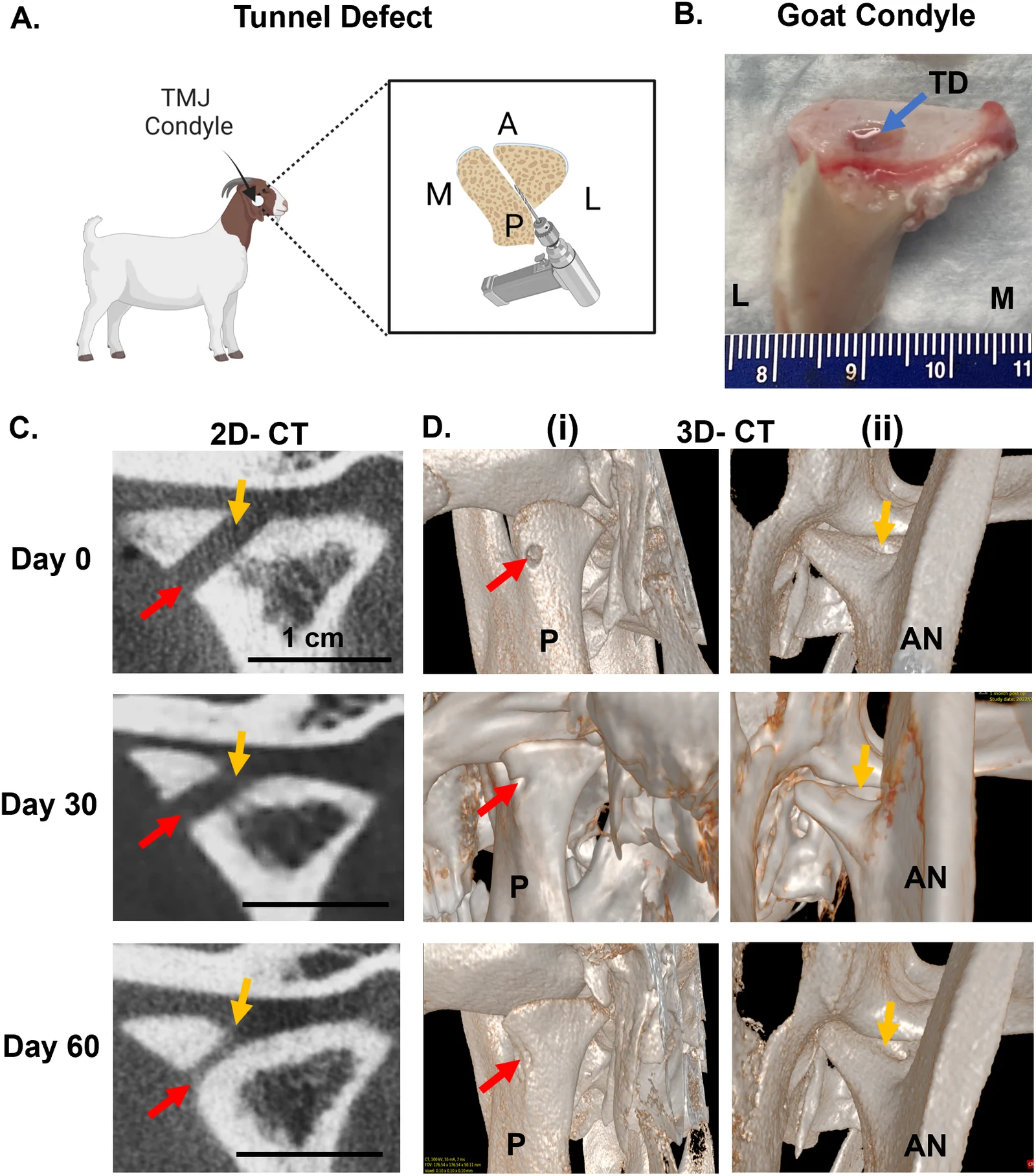
Tunnel defect in a goat TMJ condyle monitored by CT imaging and gross morphology. (A) Schematic representation of the tunnel defect (TD) surgically generated in the TMJ condyle of adult female goats. The diagram shows the alignment and positioning of the drill bit to generate tunnel defects. Key anatomical landmarks are labeled to provide a reference for the defect’s location. (B) Representative digital photograph showing gross morphology of the TMJ condyle 60 days after creation of the tunnel defect. The blue arrow indicates the TD on the condylar surface. (C-D) Representative 2D images (coronal view) and 3D reconstruction of CT scans illustrating the location and incomplete healing of the TD at the surface and posterior aspect of the condyle. Posterior and anterior aspects of the condyle were evaluated in the 3D CT images. Yellow arrows highlight the location of the defect on the condylar surface in both 2D and 3D CT images at different time points (0, 30, 60 days). In contrast, red arrows highlight the OCD in the posterior aspect of the TMJ condyle at 0-, 30-, and 60 days post-surgery. Scale bars are 1 cm. Medial (M), lateral (L), anterior (AN), posterior (P).

In this second group, a 3.2 mm through-hole tunnel was drilled from the posterior-inferior-lateral aspect of the condyle in a superior-anterior direction to the mid-condylar surface (Fig 2A). First, the TMJ was accessed as in the superior defect. Next, a 1.2–2.0 cm superior-inferior incision was made from the center of the TMJ incision over the condyle, and the muscle was separated. Once the bone was clearly accessible, a 3.2 mm bone drill bit (Medtronic) was rested on the bone surface and visually angled towards the surface of the condyle. A stainless steel spatula was placed under the disc to avoid damage from drilling through the hole. The disc attachments were preserved (other than the lateral attachments), unlike the superior defect. The incisions were then closed as for the superior defect using resorbable sutures.

The main steps of the surgical procedure used to create these two types of OCDs in the TMJ condyle are illustrated in S1 Fig.

### Postoperative care

After recovery, the animals were returned to their housing unit and had free access to food and water ad libitum. Postoperative monitoring included indicators of pain or discomfort, such as reduced appetite, abnormal vocalizations, withdrawal from social interaction, or signs of depression. Gait abnormalities and general lethargy were also assessed. Goats were provided with palliative care for 3 days post-operation with injections of Banamine 1.1 mg/kg IM SID. They were monitored daily for signs of pain, fever, infection, and lethargy. Body weight was recorded at 4 different time points: (i) before the day of the surgery or the same day of surgery, (ii) within 7 days post-surgery, (iii) 30 days, and (iv) 60 days after the OCDs were created.

### Analysis of computed tomography (CT Scan)

The TMJ condylar OCDs were imaged using in vivo high-resolution computed tomography (CT) on the VIMAGO GT30 system at a voxel size of 200 µm. The scan was performed using the following parameters: 360° rotation, 720 frames captured at 10 Hz, with an exposure duration of 6 ms at 100 kV and 55 mA. A three–dimensional (3D) reconstruction of the TMJ condyle was conducted using VoxelVu–Epica software. A two-dimensional (2D) simulated X-ray was created, aligning the plane of view with the defect axis to visualize the defect in a plane that best shows its size, shape, and orientation.

### OARSI macroscopic evaluation

After careful dissection, macroscopic evaluation of the TMJ condyles was conducted using the Osteoarthritis Research Society International (OARSI) scoring system, following recommendations for gross macroscopic tissue assessment, as outlined by Embree et al. [16] (S1 Table). A total of three calibrated observers scored macroscopic changes in the articular surface of the biopsied TMJ condyles at 60 days post-surgery and in unoperated controls using de-identified digital images (blinded to OCD type, though apparent in unoperated controls with no defect). The analysis was conducted across the entire condyle and the surrounding area (excluding the OCD itself) on the condylar surface.

### Histological analysis and semi-quantitative evaluation

TMJs were fixed in 10% neutral buffered formalin for 48 hours, decalcified with RDO-GOLD (4–5 days), paraffin-embedded, and sagittally sectioned at 8 µm thickness for histological examination of the regenerated joint structure and surface. Histological evaluation of tissue architecture within and surrounding the OCDs, the characteristics of the repair tissue formed within the defects, and changes in the subchondral bone underlying and adjacent to the defects were performed using hematoxylin and eosin (H&E) staining. Safranin-O/Fast-Green staining was utilized to evaluate cartilage—proteoglycans and glycosaminoglycans (GAGs)— with particular emphasis on the TMJ joint surface. Movat’s pentachrome staining was used to detect new bone and cartilage formation within the OCDs. Images were captured using a Zeiss AxioCam mounted on a Zeiss Axioskop A1 microscope, with ZEN software for analysis. For each staining, two to three sections per goat/ OCD type or control group were analyzed (n = 3 goats/group). Microscopic evaluation of the TMJ condyles was performed using the modified OARSI scoring system for histological assessments of TMJ-OA in the areas near the OCD (S2 Table) [16]. Three observers scored the same set of sections three times, with a three-week gap between the assessments. The observers blindly assessed OARSI histological scores based on Safranin O staining and structural scoring criteria, using comparable histological sections (per goat/OCD type). Sections were therefore selected in which the OCD was clearly visible, ensuring that the analyzed sections accurately captured the region of interest in each animal. OARSI histological scores from all observers were averaged per goat, with Safranin O staining and structural scoring criteria analyzed separately. Movat’s pentachrome staining provided distinct coloration of collagen, proteoglycans, and other extracellular matrix components, allowing clear distinction among bone, new bone, and cartilage.

### Statistical analysis

OARSI macroscopic scores for each goat were calculated as the mean of the three observers’ ratings. Each observer’s score was derived from the average of their three independent repeated measures made on different days. Differences between the control (no defect) and OCD groups were analyzed using the non-parametric Mann-Whitney U test. Macroscopic OARSI scoring was performed on all animals in each group (Control n = 3; SD n = 4; TD n = 6). Histological analysis, including Safranin O staining and structural OARSI scoring, was performed on a randomly selected subset of 3 animals per group. OARSI microscopic scores were compared between groups using a one-sided exact Mann–Whitney U test, reflecting our priori hypothesis that OCDs OARSI scores would exceed control scores. Exact p-values are reported throughout, with statistical significance considered at p ≤ 0.05. Differences in goat body weight across time (i) before the day of the surgery or the same day of surgery, (ii) within 7 days post-surgery, (iii) 30 days, and (iv) 60 days after the defects were analyzed using ANOVA. Statistical calculations were performed using GraphPad Prism 10, and a two-tailed p-value ≤ 0.05 was considered statistically significant.

## Results

### Animals

To evaluate the effects of surgical intervention and the creation of OCDs on general health, the body weight of each goat was monitored at four time points: before surgery (baseline), within the first 7 days post-surgery, at 30 days, and at 60 days post-defect induction. Monitoring body weight at these intervals provided insight into the animals’ recovery and overall health, revealing that the goats tolerated the surgical procedure well, with no adverse effects. On average, the animals’ body weights on the day of surgery were variable (low: 39 kg; high: 62 kg) but within the typical range for their species, sex, breed, and age. Seven days after surgery, 5 out of 10 goats experienced a loss in weight of 2.2 ± 1.5 kg (S2 Fig). Nevertheless, half of the animals surpassed their initial body weight 60 days after surgery. The other half of the animals showed substantial recovery, with body weights returning to near-baseline levels. Among the remaining animals, the average weight loss at 60 days post-surgery was 1.5 ± 1.4 Kg. However, no significant difference was observed between the animals’ baseline body weight and the weight measured 60 days following the surgery. No behavioral changes were observed in any of the goats during the entire study.

### Gross morphological assessment of TMJ condyles following surgical induction of osteochondral defects

We examined the overall appearance of the condyle to identify structural OA-like changes or signs of regeneration in our OCD goat models. TMJ condyles were harvested after 60 days to assess the extent of spontaneous healing over a clinically relevant period. On gross examination, the defect aperture remained open after 60 days, suggesting minimal cartilage repair in both OCD types (Figs 1 and 2). In each OCD group, OA-like changes were evident, and defects remained depressed and covered with fibrous tissue. Arrows showed the defect created in the mandibular condyle (Figs 1B and 2B).

### Live CT scan evaluation

The live CT scan on Day 0 (immediately following surgery) demonstrated successful creation of a distinct tunnel-shaped OCD involving the articular cartilage, subchondral bone, and cortical bone (Fig 2C). At days 30 and 60, the tunnel defect in the goat condyle showed incomplete bone regeneration (Fig 2C). The depth of the OCD varied slightly between condyle samples, likely due to the manual nature of defect creation. Like the tunnel defect, the superior defect also showed incomplete subchondral bone regeneration, with the defect sites still clearly distinguishable from the surrounding native tissue (Fig 1C). The superior defect suggested faster bone healing than the tunnel defect, likely due to its smaller size. The 3D reconstructions of the condyles further supported the findings observed in the 2D images (Figs 1D and 2D). These observations indicate that spontaneous healing of the TMJ condyle is limited within this timeframe, emphasizing the need for interventions to promote effective cartilage and bone repair.

### Histology

**Fig 3:**
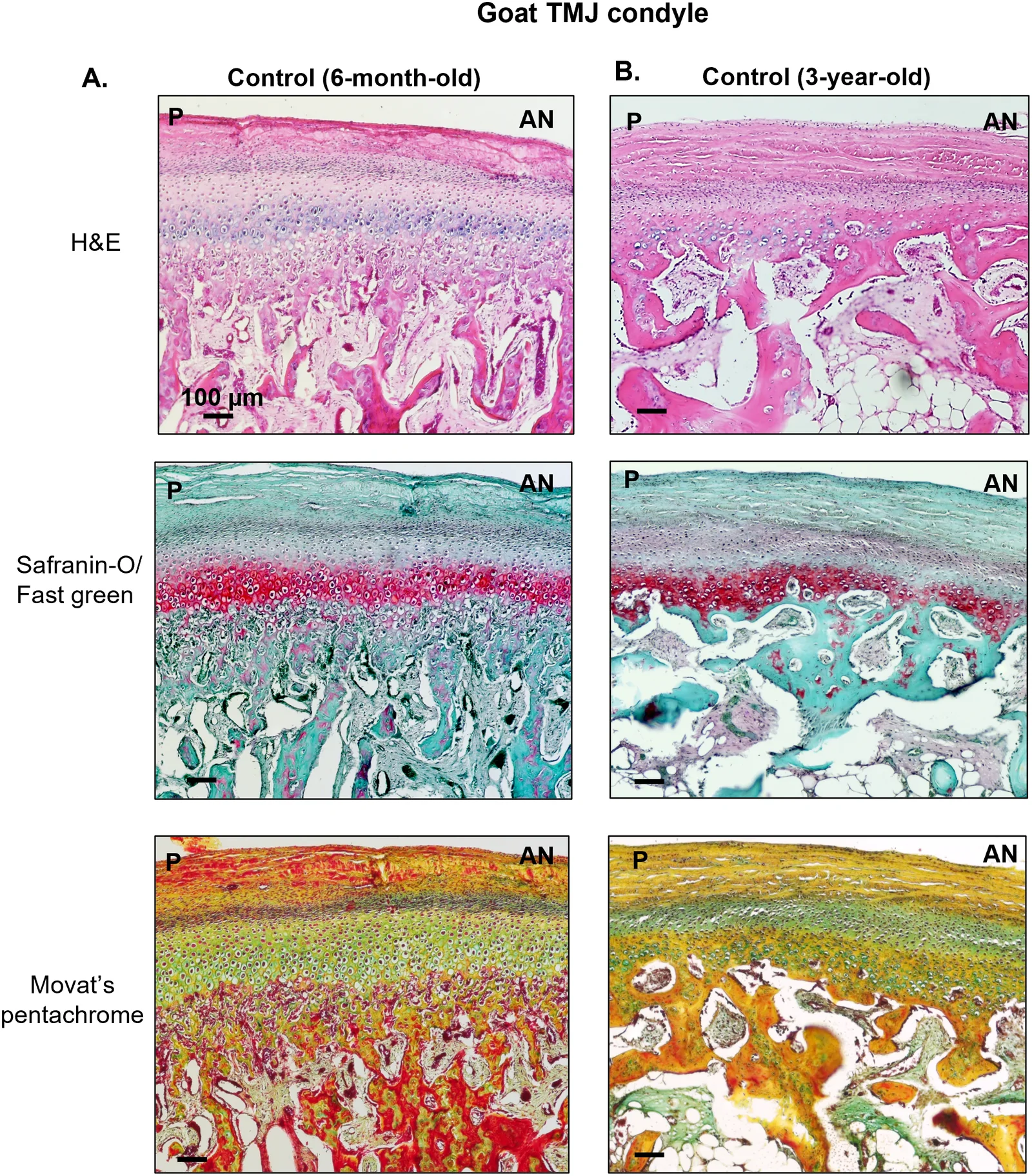
Histological architecture of goat TMJ condyles under control conditions. Representative sagittal sections from a 6-month-old (A) and a 3-year-old (B) condyle stained with H&E, Safranin-O/Fast Green, and Movat’s pentachrome. Images illustrate age-related differences in cartilage matrix and bone structure, serving as baseline morphology for comparison with defect models.

To complement the gross morphological observations, the TMJ condyles were examined histologically with H&E, Safranin-O/Fast Green, and Movat’s pentachrome staining, enabling evaluation of cellular organization and proteoglycan distribution. In addition, an in-depth analysis of the defect’s size and depth (including the full thickness of the cartilage and underlying bone) was performed to assess the defect’s potential for spontaneous healing. To account for age-related histological differences in TMJ cartilage organization, OCD groups were compared with age-matched native control condyles. This approach was necessary given the known variations in TMJ cell layer thickness, cellularity, and matrix composition between younger goats (Fig 3A) and mature 3-year-old animals (Fig 3B). Age matching to skeletally mature goats enabled a more accurate assessment of defect-associated changes by minimizing confounding effects of maturational differences in TMJ cartilage and subchondral bone structure (Fig 3). The condylar cartilage layers in the native group were observed using both H&E and Movat’s pentachrome staining, with Movat staining providing a clearer delineation of a fibrous layer (yellow), the proliferative zone (dark area under the fibrous layer), the mature zone (green), and the hypertrophic zone (green) (Figs 3 and S3). The Safranin-O images showed the presence of GAG in the control condyles (red) (Fig 3). Tissues from adult goats were compared with those from 6-month-old goats obtained from a local abattoir.

**Fig 4:**
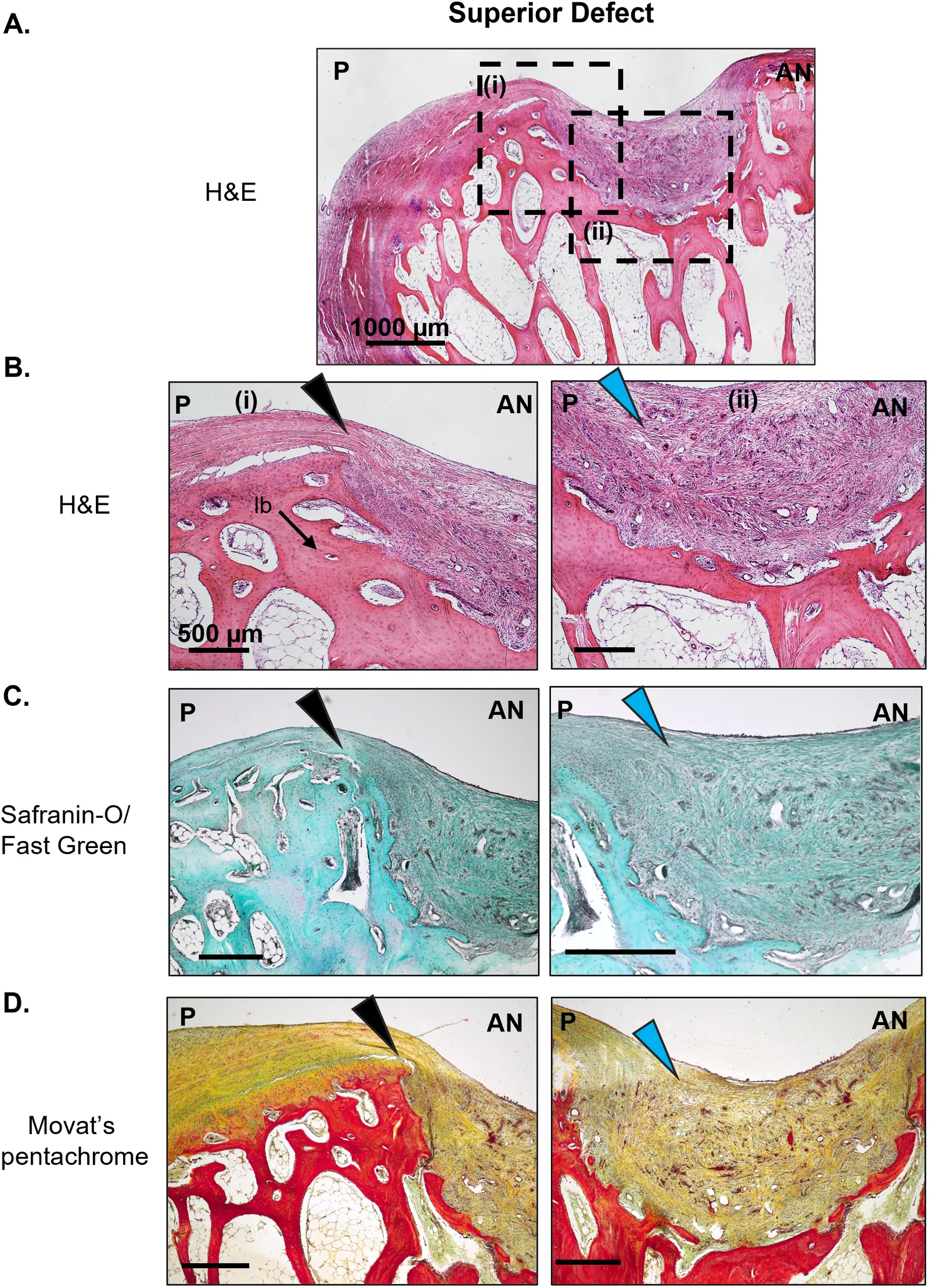
Histology of the TMJ condyles shows fibrous tissue infiltration in the superior defects, with degenerative changes in the surrounding articular fibrocartilage, marked by loss of the fibrous, transitional, and cartilaginous layers. Representative sagittal sections of TMJ condyles at 60 days post–superior defect creation, stained with (A-B) H&E, (C) Safranin O/Fast Green, and (D) Movat’s pentachrome. Panel B presents higher-magnification H&E images of regions A(i) and A(ii), as indicated by the dotted boxes. Histological staining confirmed infiltration of the superior defect with fibrous tissue (blue arrowheads). Degeneration of the articular fibrocartilage surrounding the defect was evident, with disruption and loss of the fibrous, transitional, and cartilaginous layers (black arrowheads). Characterization of the condyle tissue using Safranin-O/Fast-Green staining revealed an absence of GAG. Posterior (P), anterior (AN), lamellar bone (lb).

**Fig 5:**
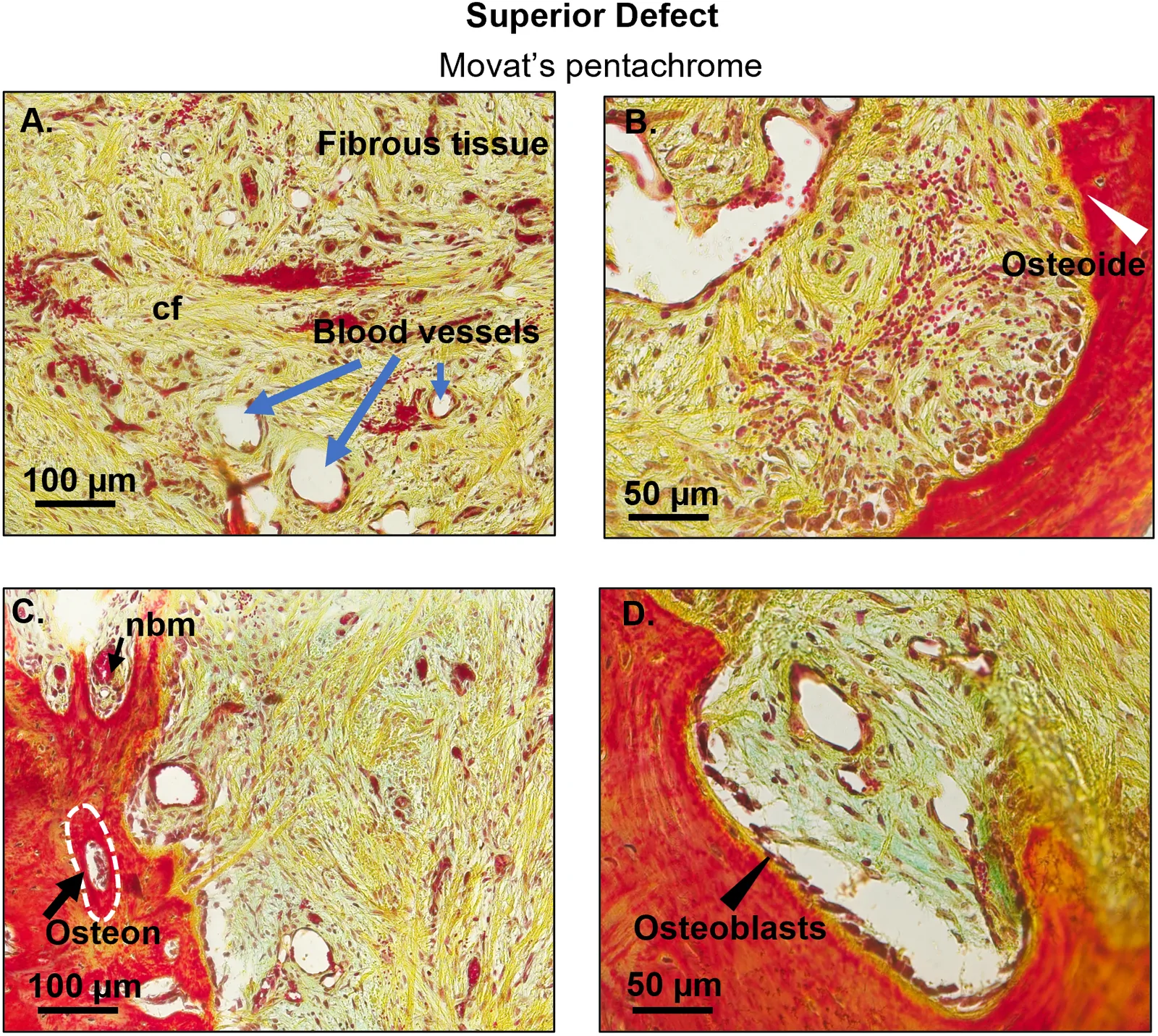
Cellular and tissue-level features of superior defects 60 days post-surgical creation. (A) High-magnification images of histological sagittal sections stained with Movat’s pentachrome revealed evidence of fibrous tissue formation and disorganized collagen fibers (light yellow staining) in the superior defect of the goat TMJ condyle 60 days post-surgical creation. (B) Osteoid formation surrounding the defect was detected adjacent to the defect margins within the subchondral bone region, where active bone remodeling occurs (white arrowhead). (C) The lower-left image shows a well-organized osteon (outlined with a white dotted line) within the subchondral bone. (D) Osteoblasts are seen along the bone surfaces surrounding the defect (black arrowhead). Collagen fibers (cf), new bone matrix (nbm).

Compared with age-matched native controls, histological evaluation revealed that the superior OCD contained predominantly fibrous tissue across all staining modalities (Fig 4A–4D). In Movat’s pentachrome–stained sections, the defect area was characterized by abundant yellow-staining collagen fibers, indicative of dense fibrous connective tissue, with minimal blue–green proteoglycan-rich matrix (Fig 4D). The fibrous tissue lacked the organized zonal architecture and chondrocyte morphology typical of native TMJ cartilage, consistent with fibrovascular repair rather than fibrocartilaginous regeneration. Staining revealed loss of the perichondrium over the OCD, a dense fibrous connective tissue layer that covers the cartilage. Furthermore, the surrounding articular fibrocartilage showed signs of degeneration, including the loss of its zonal structure, which is characterized by fibrous, transition, and cartilaginous layers (Fig 4D) (for normal anatomy, see Fig 3B). The Safranin-O/Fast-Green staining further confirmed that the tissue was fibrous and that there was no GAG at the defect site and on the surface surrounding the defect, indicating the absence of new cartilage formation within the OCD (Fig 4C). Under higher magnification, the presence of disorganized collagen fibers was visualized in the superior defect (Fig 5A–5D). Representative images highlight the predominance of fibrous tissue within the central and basal regions of the superior defect. In addition, blood vessels were readily observed within the defect area, indicating fibrovascular tissue infiltration rather than mature cartilaginous repair. The presence of vascular structures further supports an incomplete regenerative response and suggests ongoing remodeling within the defect site. Histological assessment using Movat’s pentachrome staining of the mandibular condyle revealed osteoblasts lining the osseous walls of the OCD and ongoing bone remodeling, as evidenced by large areas of osteoid (Fig 5).

**Fig 6:**
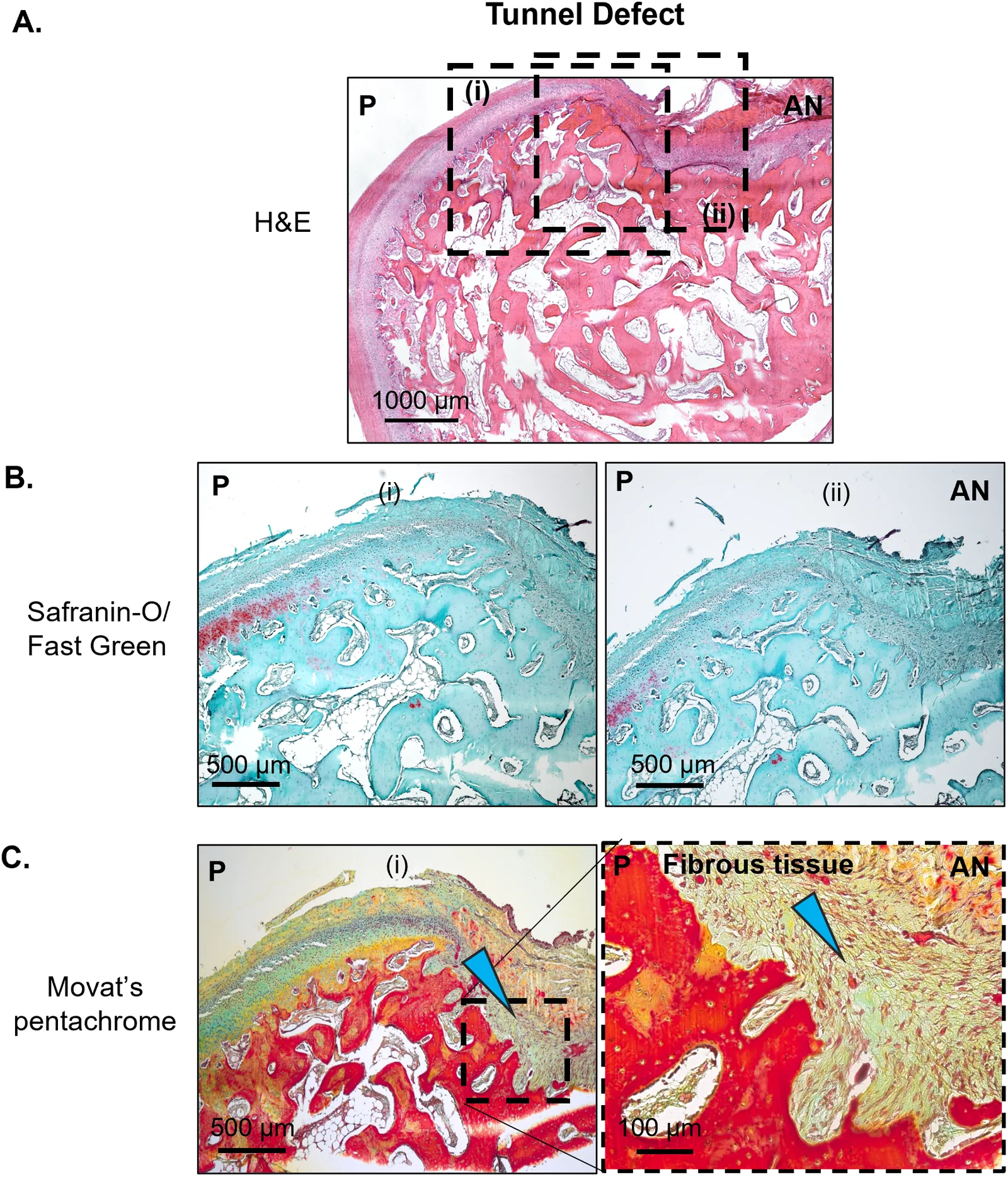
Tunnel defects were consistently infiltrated with fibrous tissue and exhibited active bone remodeling within the subchondral region, indicating a reparative but incomplete healing response 60 days post-surgical creation. Representative sagittal sections of TMJ condyles at 60 days post–tunnel defect creation, stained with (A) H&E, (B) Safranin O/Fast Green, and (C) Movat’s pentachrome showing incomplete healing of the OCD. Panels B(i) and B(ii), as well as C(i), are higher-magnification views of the areas indicated by rectangles in A(i) and A(ii). Panel C(i) shows a dotted box and, on the right, a corresponding higher-magnification image illustrating detailed features of the tunnel defect. The Movat’s pentachrome staining allowed us to confirm that all defects were infiltrated with fibrous tissue (blue arrowhead). Characterization of the condyle tissue using Safranin-O/Fast-Green staining revealed an absence of GAG. No new cartilage was detected at the defect site. However, new bone was visualized (bright red staining) surrounding the OCD. Posterior (P) and anterior (AN) aspects of the TMJ condyle.

**Fig 7:**
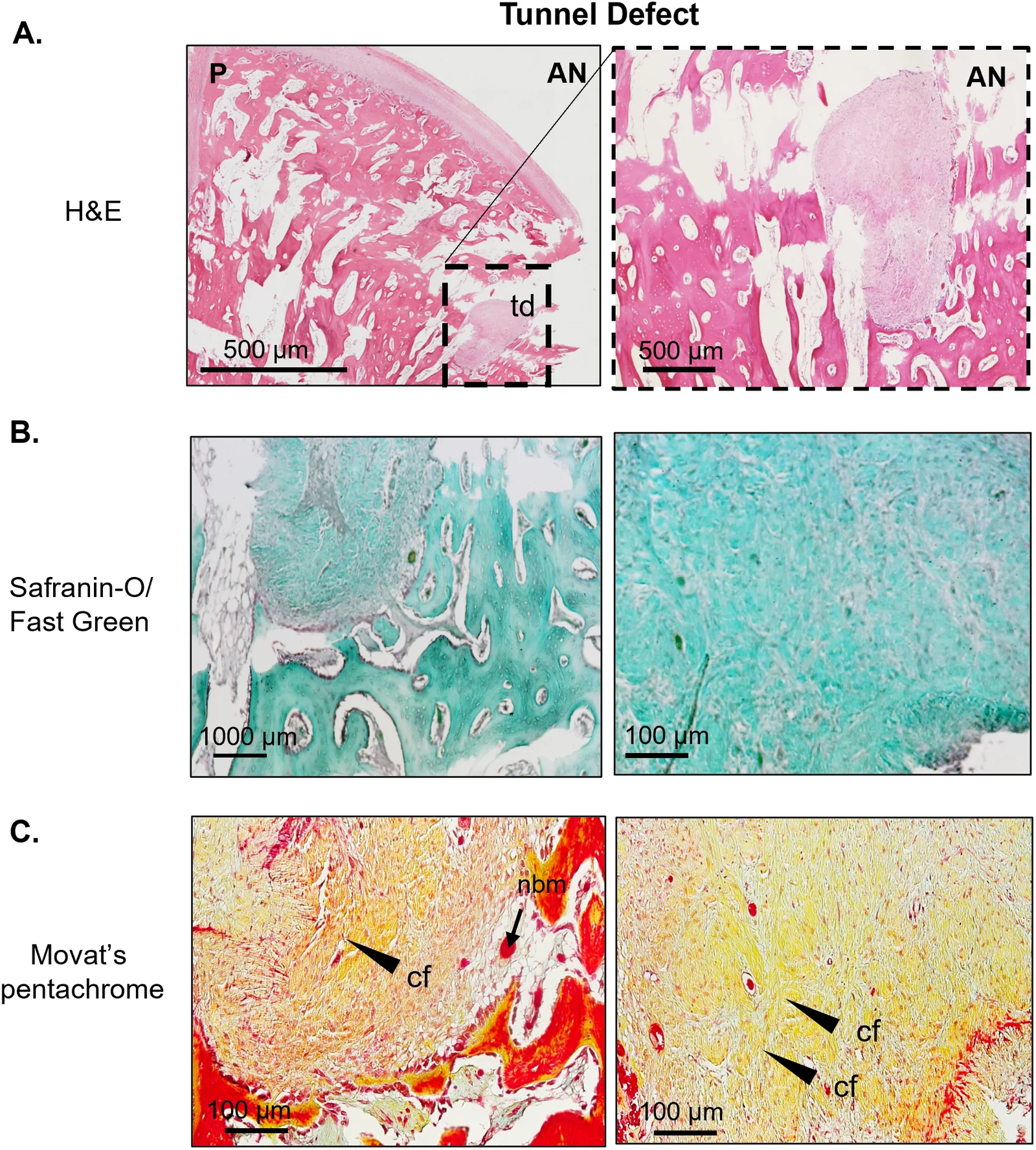
Histology of the tunnel defect in the subcondylar bone region shows limited structural restoration despite localized bone remodeling. Representative sagittal sections of TMJ condyles at 60 days post–tunnel defect creation, stained with (A) H&E, (B) Safranin-O/Fast Green, and (C) Movat’s pentachrome. The tunnel defect showed a fibrous core surrounded by a large amount of newly woven bone, which reduced the size of the bone defect. The edges of the defect (black arrowhead) indicate early stages of bone repair, though full regeneration has not occurred. Additionally, Movat’s pentachrome allows visualization of new bone matrix (nbm) surrounding the walls of the defect (black arrows) and collagen fibers (cf) (black arrowhead). Panel A(ii) provides a higher-magnification view of the region outlined by the dotted rectangles in A(i). At 60 days post–defect creation, the subchondral tunnel defect remains clearly identifiable, indicating incomplete spontaneous repair. Posterior (P) and anterior (AN) aspects of the TMJ condyle; tunnel defect (td).

Similarly, the tunnel OCDs did not exhibit healing of the cartilage defect. However, they showed a more stratified tissue architecture than that of the superior OCD, with multiple layers of connective tissue forming around the defect (Fig 6A–6C). In addition, Safranin-O staining showed a substantial reduction in cartilage proteoglycan content at the defect boundaries and the surface of the entire OCD (Fig 6B). Due to the orientation of the tunnel defect in the TMJ condyle, only a small portion of the tunnel was visible in each sagittal section, requiring histology at the surface (Fig 6) and somewhere along the tunnel (Fig 7). Even though H&E staining allowed for clear visualization of the cellular details of the condylar cartilage, it was less effective than Movat’s in distinguishing mineralized from non-mineralized tissue components. However, H&E images clearly showed the formation of woven bone around the tunnel (Fig 7) and areas of lamellar bone beneath the surface of the superior defect (Fig 4B).

### OARSI macroscopic scoring

**Fig 8:**
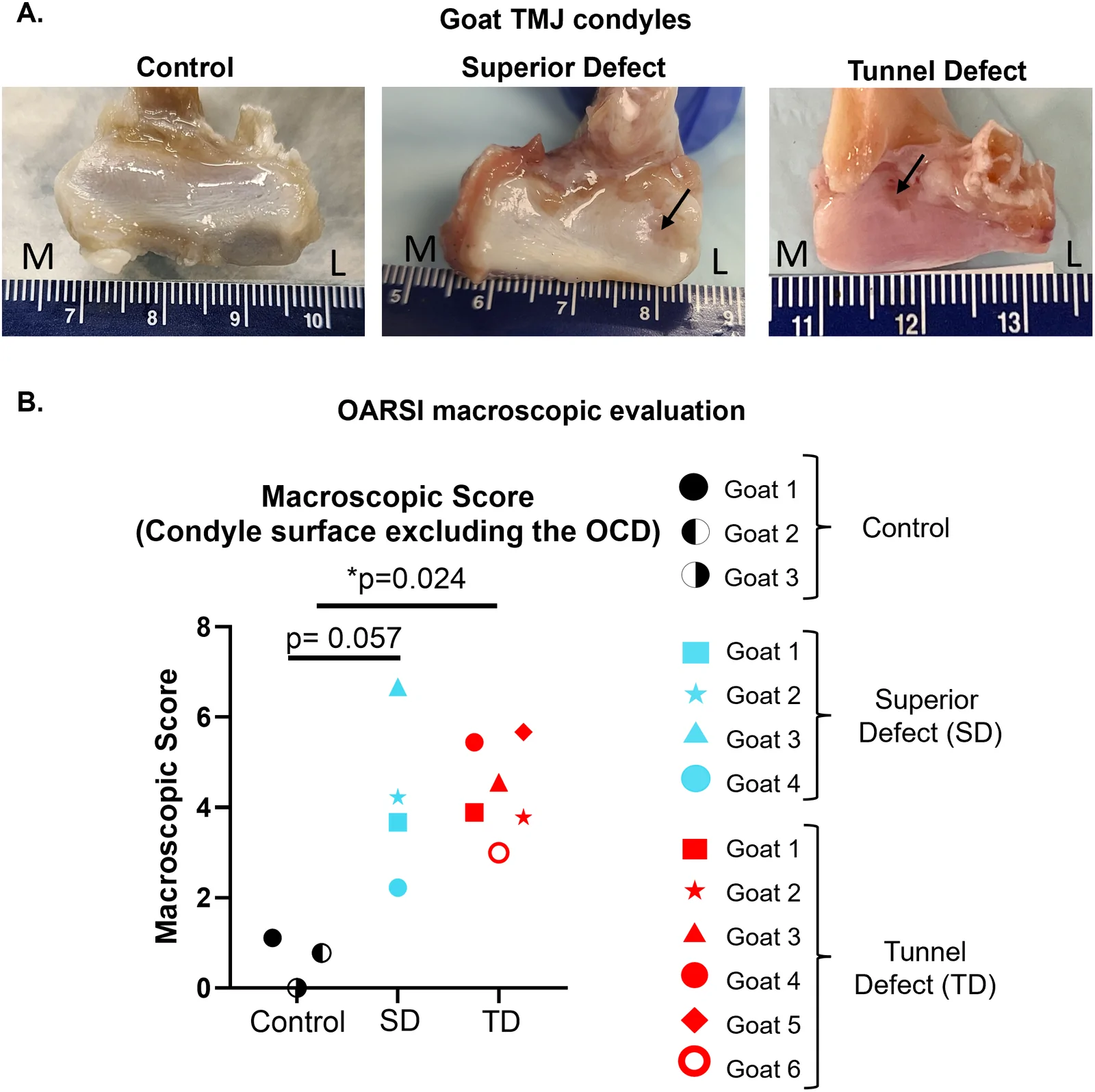
OARSI macroscopic evaluation of the goat TMJ condylar surface (excluding the osteochondral defect) 60 days after defect creation. (A) Representative gross morphology of TMJ condyles from control and TMJs with superior and tunnel defects. (B) Macroscopic scoring of control (n = 3), superior defect (SD) (n = 4), and tunnel defect (TD) (n = 6) groups across multiple goats per group, analyzed using the OARSI scoring system. Statistical significance was defined as *p < 0.05.

To further confirm the OA-like changes in our model, we used the OARSI recommendations for a macroscopic and histological scoring system [16]. The macroscopic scoring system assesses alterations in cartilage surface integrity, such as fissures, erosion, and loss of cartilage. OARSI macroscopic scores for the experimental condyles ranged from 2 to 6, indicating surface irregularities and erosion (Fig 8). No differences were found between the superior defect group and the control group, given the limited sample size. However, the trend toward higher scores suggests that the superior OCD initiated OA-like degenerative changes. The tunnel defect group showed statistically significant differences in OARSI scores compared with the control group, suggesting that tunnel OCD caused substantial cartilage damage (Figs 8 and S4). It is important to note that the OCD was not factored into the OARSI scoring, only the articular surface surrounding the OCD (Fig 8). To assess whether OA-like changes were confined to the region immediately surrounding the OCD, we analyzed both the defect-adjacent area (S4 Fig) and the entire condylar surface, excluding the defect itself (Fig 8). Similar degenerative changes were observed in both regions, indicating that OA-like alterations extended across the entire condylar surface rather than being localized to the defect site (S5 Fig).

### OARSI microscopic scoring

**Fig 9:**
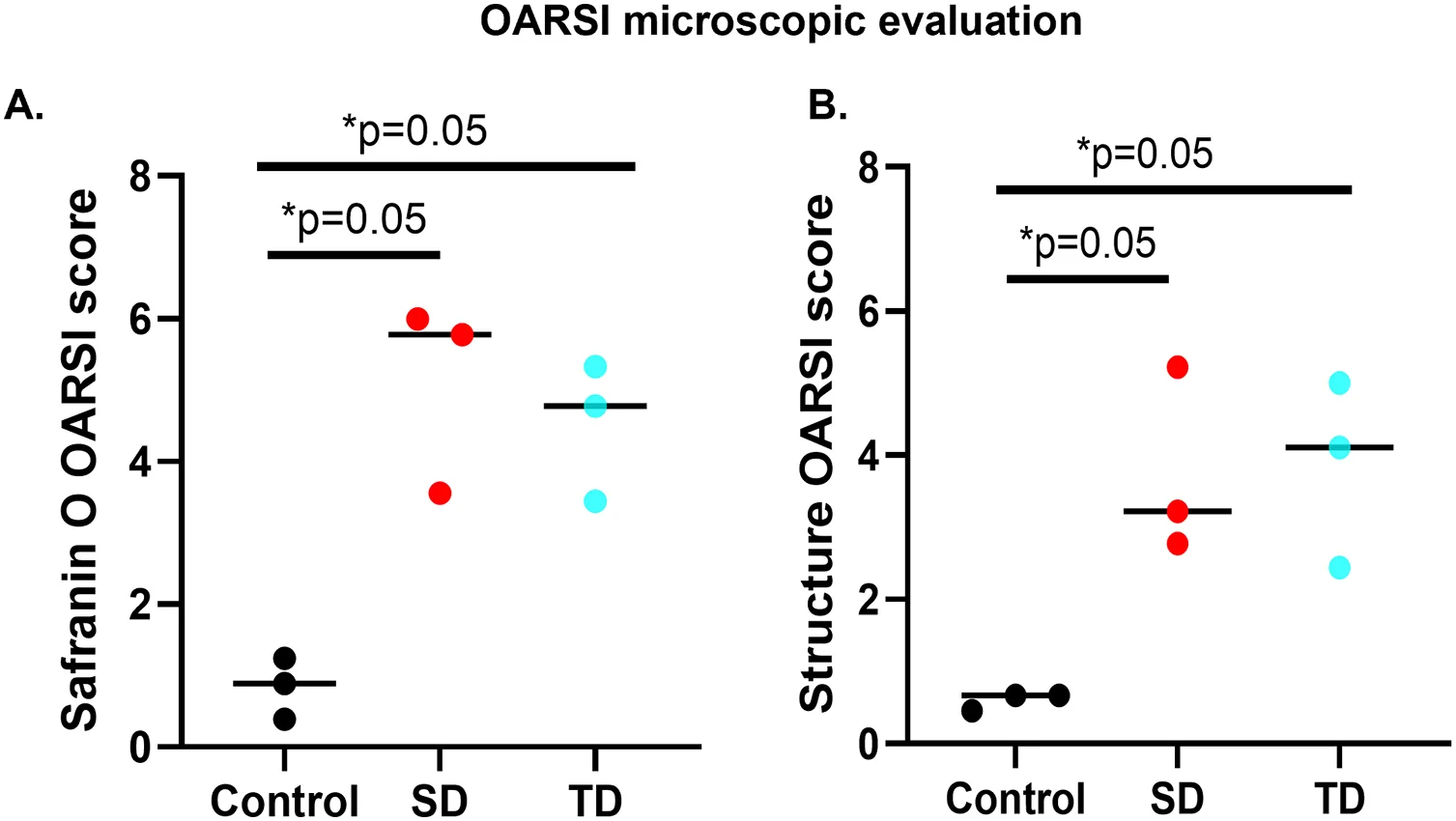
OARSI microscopic evaluation of goat TMJ condyles 60 days post-defect creation. Microscopic scoring was performed using the OARSI system to assess cartilage and subchondral bone changes in control and defect groups 60 days after generating the superior defect (SD) and tunnel defect (TD). Statistical analysis was conducted using the non-parametric one-sided exact Mann–Whitney U test to compare groups. (A) Comparison of microscopic Safranin O scores between control, SD and TD groups across multiple goats per group (n = 3 goat/group). (B) Comparison of microscopic Structure scores between control, SD and TD groups across multiple goats per group (n = 3 goat/group). Statistical significance was defined as *p < 0.05.

OARSI microscopic evaluation revealed histological alterations in TMJ condyles with surgically induced OCDs compared with age-matched native controls. Both superior defects (SD) and tunnel defects (TD) exhibited a marked increase in Safranin-O scores, indicating loss of proteoglycan content relative to control condyles (*p = 0.05) (Fig 9A and 9B). The Safranin-O scores fall within the higher range of the scale, specifically scores corresponding to loss of proteoglycan staining in the upper two-thirds to the entire thickness of the fibrocartilage (scores ~3–6). Similarly, statistically significant differences in OARSI structure scores were observed between defect groups and controls for both defect types, suggesting that matrix composition was substantially compromised and that structural changes in cartilage occurred at this time point. For the structure parameter, the predominant scores were in the mid-range (scores 2–5), reflecting surface irregularities and occasional fissuring, but no extensive erosion or full-depth cartilage loss. OCD microscopic scores were higher than those of the control group for all observations, resulting in complete rank separation between groups. The one-sided Mann–Whitney U test showed a borderline significant difference between groups, with OCD greater than control (U = 9, p = 0.05).

Together, these findings indicate early OA-like changes characterized primarily by proteoglycan depletion rather than severe structural degeneration of the TMJ cartilage.

## Discussion

We have developed a new OCD model in goats using a retrograde drilling approach and compared outcomes with those of the conventional anterograde drilling method. Both defects proved critically sized, with no spontaneous cartilage healing and incomplete subchondral bone regeneration within 60 days post-surgery. The fibrocartilage surrounding both defect types underwent OA-like degeneration. However, the tunnel OCD provided a larger defect size, preservation of the disc attachments, less manipulation of the TMJ, and more reproducible OA-like changes per the OARSI scoring, compared to the superior defect, but required greater surgical precision during drilling. As a result, this model offers a more feasible and consistent alternative.

The tunnel defect approach differs substantially from previously reported goat models developed to investigate regenerative therapies for OCDs. Generally, prior OCDs have been relatively small in size compared to clinically relevant TMJ lesions [22,24]. As a result, the observed regenerative outcomes may overestimate repair potential and limit the model’s applicability for evaluating implant performance in larger, human-scale defects. Therefore, we proposed that increasing the defect size by performing the tunnel defect would accelerate TMJ degeneration, thereby reducing the experimental timeframe required for cost-effective drug screening and evaluation of implant-based tissue regeneration strategies. Nevertheless, when creating the tunnel defect, a small variance in the drill angle led to a larger variance in the location of the cartilage defect due to the retrograde approach, especially since a larger drill bit could not be inserted over the condyle to drill in a superior-inferior direction. Conversely, when a superior defect was executed, it was consistently located in the same area. However, the depth of the superior defect is variable, as we could not use drill bit depth stops because the constrained angle of approach would damage the disc and articular surface. Another drawback of the superior OCD technique is that it involves pulling one-half of the disc medially to expose the condylar surface. This step could potentially affect the integrity of the disc and lead to disc pathologies. Nonetheless, the goats with superior OCDs showed no alterations in behavior, eating difficulties, or notable weight loss following the surgery, indicating the absence of TMJ disc pathology.

Consistent with our study, Zhu et al. [22] created a superior OCD within the central region of the mandibular condyle, measuring 3 mm in diameter and 5 mm in depth, and by 6 weeks following defect creation, untreated defects showed limited regenerative capacity. This was characterized predominantly by fibrous, non-cartilaginous tissue and inflammatory changes within the defect area, along with moderate disruption of structural integrity. However, previous in vivo studies using conventional superior OCDs in the TMJ condyle were performed in male goats. In contrast, our study utilized female goats, given the higher prevalence of TMJ-OA reported in females than in males [28,29]. This choice enhances the clinical relevance of the model, providing a more accurate preclinical setting for evaluating regenerative therapies targeting TMJ-OA.

Another important distinction between previous studies and our current work is that, although untreated OCDs demonstrated limited spontaneous healing and incomplete cartilage regeneration, they did not report formal osteoarthritis grading scores [24]. Additionally, prior studies used primarily younger goats (6–28 months) [22,24]. Our work targeted 3- to 4-year-old animals to more effectively recapitulate the diminished repair capacity observed in mature joints and to reduce the influence of spontaneous healing, which is more pronounced in younger animals.

While both the superior and tunnel defects can generate critically sized lesions needed to study regenerative approaches, the tunnel defect offers the added advantage of precision when combined with a surgical guide, allowing more accurate control of drill orientation. As a result, the second model offers a more feasible and consistent alternative.

OCD models specific to the TMJ are required because the TMJ is unique among diarthrodial joints. Although neuromuscular and musculoskeletal disorders affecting the TMJ and jaw function are quite prevalent, the TMJ has not been investigated as extensively as other diarthrodial joints, such as the knee [30,31]. The TMJ is a synovial joint, like the diarthrodial joints found in the appendicular skeleton, but with key anatomical and functional differences. From a developmental perspective, the knee joint (KNJ) originates from the mesoderm, whereas the TMJ is derived from ectodermal cranial neural crest cells [32]. The two joints also differ in cartilage type: the KNJ contains hyaline cartilage, predominantly composed of Type II collagen, and the TMJ contains secondary fibrocartilage, which is enriched in Type I collagen [32]. Functionally, the TMJ exhibits both rotational and translational movements, with the condyle capable of sliding anteriorly over the articular eminence, a large translation not observed in the knee [33]. In addition, unlike the relatively stable menisci of the knee, the TMJ features a mobile disc susceptible to displacement, contributing to a range of TMDs [34].

Procedures typically used to repair hyaline cartilage in appendicular joints, including microfracture, mosaicplasty, autologous chondrocyte implantation (ACI), and matrix-induced ACI (MACI), have not been attempted for the regeneration of mandibular condylar cartilage [35]. These procedures are likely to present limitations when applied to the TMJ condyle, given its distinct anatomical features, biomechanical environment, and functional requirements [32,33]. In standard ACI/MACI procedures for the knee, chondrocytes are harvested from a non-load-bearing area of the same joint, ensuring compatibility with the joint’s mechanical and biological environment. However, the TMJ lacks a comparable non-weight-bearing cartilage surface from which viable chondrocytes can be harvested. Moreover, autologous osteochondral transplants from other diarthrodial joints raise concerns about proper biomechanical function, adequate graft integration, and tissue homeostasis, given the significant differences in tissue composition, architecture, and functional loading between TMJ fibrocartilage and hyaline cartilage. For example, TMJ cartilage is adapted to withstand shear and tensile forces, while knee cartilage is specialized for resisting compressive loads. This biomechanical mismatch means that tissues engineered for knee cartilage may fail under the unique multidirectional loading patterns of the TMJ, leading to poor durability or degeneration. Therefore, alternative approaches specific to TMJ condylar cartilage regeneration have yet to be established.

While the OCDs described here induce cartilage and subchondral bone changes, they do not necessarily reproduce the gradual, multifactorial progression characteristic of naturally occurring TMJ-OA. Natural TMJ-OA develops through a complex, multifactorial process involving mechanical, biochemical, and inflammatory pathways that unfold gradually over time, whereas the OCDs described here produce a localized injury with a distinct and likely faster progression. As such, this model should currently be regarded as a reproducible platform for evaluating the efficacy of regenerative therapies on a defined OCD, rather than a validated surrogate for natural TMJ-OA pathogenesis. As such, findings from this model should be interpreted with caution when extrapolating to the pathophysiology of natural disease progression.

Another limitation of this study is the use of unoperated controls rather than sham-operated controls. While unoperated controls allowed us to characterize baseline TMJ morphology and histology, they do not account for potential confounding effects of anesthesia, surgical exposure, and incision-related inflammation that may independently influence the joint tissue response. Future studies incorporating sham-operated controls would allow more precise attribution of the observed changes specifically to the OCD itself, rather than to the surgical procedure more broadly. We would also like to highlight that, despite this limitation, the unoperated control joints in this study provided valuable baseline histological data. Detailed histological characterization of the aged goat TMJ is currently limited in the literature, and we believe the baseline histological data presented here help address this gap by providing a normative reference for TMJ condylar cartilage and subchondral bone architecture in this large-animal model.

Considering histological scoring is the gold-standard OARSI outcome for cartilage repair/degeneration studies, using the Safranin O OARSI score data from the current study, we performed post hoc power analyses to estimate the sample size required to detect a 50% reduction in OARSI score with a therapeutic intervention, assuming α = 0.05 and 80% power. Sample sizes were initially calculated for a two-sample t-test and inflated by a factor of 1.15 to account for the reduced efficiency of the Mann–Whitney U test, which is appropriate for ordinal OARSI score data. Based on this analysis, a sample size of 7 animals per group would be required for a study using the Superior Defect (mean ± SD = 5.11 ± 1.35; Cohen’s d = 1.89), or 6 animals per group for the Tunnel Defect (mean ± SD = 4.52 ± 0.97; Cohen’s d = 2.33).

## Conclusions

In summary, this study establishes a practical and reproducible goat model of OCD with OA-like degeneration for testing and refining future innovative regenerative therapies targeting focal lesions of TMJ cartilage.

## Supporting information

S1 Fig Digital pictures illustrate key aspects of the surgical procedure to generate OCDs in the goat TMJ condyle. (A) Exposure of the condyle surface and retraction of the TMJ disc medially to create the superior defect. (B) A 5 mm deep and 2.4 mm wide hole (superior defect) was drilled from the superior-lateral aspect to the inferior-medial aspect of the TMJ condyle. The blue arrowhead points to the condylar surface where the defect was drilled during surgery. (C-D) A tunnel defect was drilled using a 3.2 mm drill bit, from the posterior-inferior-lateral aspect of the condyle in a superior-anterior direction to the mid-condylar surface. To create the tunnel, a periosteal elevator was inserted between the surface of the condyle and the disc to avoid damage to the articular disc. The yellow arrowhead points to the posterior region of the condyle where the defect was drilled during the surgery.
(TIF)

S2 Fig Changes in goat body weight over the experimental period. Mean body weight (kg) of goats subjected to (A) TMJ superior defect (n = 4) and (B) tunnel defect (n = 6) surgeries, measured at four time points: pre-surgery, within 7 days post-surgery, and at 30 and 60 days following surgical induction of OCDs in the TMJ condyle. (C-D) Body weight remained stable across all post-surgical time points, with no statistically significant differences relative to pre-surgery measurements. These results indicate that surgical interventions did not significantly affect overall body weight, suggesting systemic health was maintained throughout the study. Data are presented as mean ± standard deviation (SD). No significant differences were observed across time points.
(TIF)

S3 Fig Movat’s pentachrome staining of control TMJ condyles highlighting specific subregions. Panel A shows the histological architecture of a 6-month-old condyle, while Panel B represents a 3-year-old condyle. Distinct zones—superficial, proliferative, pre-hypertrophic, and hypertrophic—are clearly visualized, demonstrating normal tissue organization and extracellular matrix composition. These images establish age-related baseline structural integrity for comparison with surgically induced defects and repair outcomes. Scale bar: 100 µm.
(TIF)

S4 Fig OARSI macroscopic evaluation of the TMJ condylar surface in goats, focusing on the area surrounding the OCD, 60 days post-defect creation. Macroscopic OARSI scoring was performed on native control condyles (n = 3) and those with superior (SD) (n = 4) and tunnel defects (TD)(n = 6). Statistical analyses were performed to compare defect groups with control samples. Statistical significance was defined as *p < 0.05.
(TIF)

S5 Fig Osteoarthritis-like changes on the TMJ condyle 60 days following osteochondral defect creation. (A) Representative macroscopic image of a goat condyle bearing a surgically created superior defect. Arrows indicate OA-like surface changes both adjacent to and distant from the defect site. The cartilage doesn’t appear entirely smooth, with some fibrillation; no exposed subchondral bone is visible in the non-defect areas. No obvious deep fissuring or full-thickness loss away from the defect consistent with early-to-moderate OA-like changes. (B) Representative macroscopic image of a condyle bearing a tunnel defect. Arrows indicate OA-like surface changes. The surrounding cartilage appears thinner or discolored in this region. The pink/red tint suggests loss of the normal articular cartilage (cartilage thinning) and possible exposure of deeper tissue or vascular invasion. Evidence of erosion of the peripheral cartilage appears to expose underlying tissue in some areas. The dotted circle highlights the location of the OCD in both images (not included in the analysis).
(TIF)

S1 Table OARSI macroscopic evaluation parameters. This table summarizes scoring parameters for gross assessment of TMJ condyle integrity following surgical defect creation.
(PDF)

S2 Table OARSI recommended histological evaluation. (PDF)

S1 Appendix Supporting data. (XLSX)
