# Supplementary material for: Goat temporomandibular joint osteochondral defects for future regenerative studies of osteoarthritis-like degeneration
Source: PLoS One. 2026 Aug 19;21(8):e0356421. doi: 10.1371/journal.pone.0356421 (PMC13489421; doi:10.1371/journal.pone.0356421)
Supplement: Supplementary file 1 [file pone.0356421.s006.pdf]

S1 Table. OARSI Macroscopic Evaluation

OARSI SCORE	
**0**	Normal
**1**	Focal surface roughness
**2**	Widespread surface irregularity
**3**	Beginning surface fibrillation (mostly intact surface. Surface appears to be slightly peeling off)
**4**	Severe surface fibrillation (75% fibrillation, extended depth through surface)
**5**	Beginning erosion (<4mm of length)
**6**	Severe erosion (>8mm length)
**7**	Slight ulceration (cartilage lost, bone fragment intact, 2mm deep)
**8**	Severe ulceration (bone surface remodeling, 5mm deep)
