# Supplementary material for: Goat temporomandibular joint osteochondral defects for future regenerative studies of osteoarthritis-like degeneration
Source: PLoS One. 2026 Aug 19;21(8):e0356421. doi: 10.1371/journal.pone.0356421 (PMC13489421; doi:10.1371/journal.pone.0356421)
Supplement: Supplementary file 2 [file pone.0356421.s007.pdf]

S2 Table. OARSI Recommended Histological Evaluation

OARSI SCORE	*Safranin O*
**0**	Uniform staining throughout articular cartilage
**1**	Loss of staining in superficial zone of hyaline cartilage <50% the length of the condyle or plateau
**2**	Loss of staining in superficial zone of hyaline cartilage 50% the length of the condyle or plateau
**3**	Loss of staining in the upper 2/3's of hyaline cartilage <50% the length of the condyle or plateau
**4**	Loss of staining in the upper 2/3's hyaline cartilage 50% the length of the condyle or plateau
**5**	Loss of staining in all the hyaline cartilage <50% the length of the condyle or plateau
**6**	Loss of staining in all the hyaline cartilage 50% the length of the condyle or plateau

OARSI SCORE	*Structure*
**0**	Normal
**1**	Surface Irregularities
**2**	Fissures in <50% surface
**3**	Fissures in 50% surface
**4**	Erosion 1/3 hyaline cartilage <50% surface
**5**	Erosion 1/3 hyaline cartilage 50% surface
**6**	Erosion 2/3 hyaline cartilage <50% surface
**7**	Erosion 2/3 hyaline cartilage 50% surface
**8**	Full depth erosion hyaline cartilage <50% surface
**9**	Full depth erosion hyaline cartilage 50% surface
**10**	Full depth erosion hyaline and calcified cartilage to the subchondral bone <50% surface
**11**	Full depth erosion hyalin and calcified cartilage to the subchondral bone 50% surface
