# Supplementary material for: Goat temporomandibular joint osteochondral defects for future regenerative studies of osteoarthritis-like degeneration
Source: PLoS One. 2026 Aug 19;21(8):e0356421. doi: 10.1371/journal.pone.0356421 (PMC13489421; doi:10.1371/journal.pone.0356421)
Supplement: Supplementary file 3 [file pone.0356421.s010.pdf]

University of Pittsburgh

Salk Hall
3501 Terrace Street
Pittsburgh, PA 15261
Phone: 412-648-3101
Fax: 412-624-6685

School of Dental Medicine
Center for Craniofacial Regeneration
Collaborative for REsearch to Advance TMD Evidence (CREATE) Center

July 16, 2026

Title:

Goat temporomandibular joint osteochondral defects for future regenerative studies of osteoarthritis-like degeneration

Authors:

Mairobys Socorro, Xudong Dong, Juan M Taboas, Alejandro J Almarza

We sincerely thank the editor and reviewer for the insightful comments and constructive feedback. We have carefully addressed each comment and revised the manuscript accordingly. We believe that the reviewers' suggestions have significantly strengthened our manuscript. The discussion has been reworked to better reflect the results.

Point-to-point response to the Academic Editor:

1. Please ensure that your manuscript meets PLOS ONE's style requirements, including those for file naming. The manuscript has been revised to comply with PLOS ONE's style requirements.

2. We note that the grant information you provided in the 'Funding Information' and 'Financial Disclosure' sections do not match. We have provided the correct grant numbers for this study in the 'Funding Information' section.

3. Please note that funding information should not appear in any section or other areas of your manuscript. We removed all funding-related text from the manuscript.

4. When completing the data availability statement of the submission form, you indicated that you will make your data available on acceptance. We have clarified that "All relevant data are within the paper and its Supporting Information files"

5. We note that Figure 1, 2, and 8; Supporting Information Figure 2 in your submission contain copyrighted images. Thank you for your careful review of our submission and for bringing this matter to our attention. We would like to respectfully clarify that Figures 1, 2, and 8, as well as Supporting Information Figure 2, are entirely original and were produced by the authors. These figures do not contain any copyrighted or third-party material, and no images were reproduced or adapted from previously published sources.

6. If the reviewer comments include a recommendation to cite specific previously published works, please review and evaluate these publications to determine whether they are relevant and should be cited. N/A

Point-by-point response to reviewer #1:

1. The title can be misleading and needs to be revised. The current title is potentially misleading and should be revised. Cartilage damage-induced changes in adjacent tissue are likely distinct from those observed in natural TMJ osteoarthritis. Furthermore, the application of this model for regenerative therapies is not directly demonstrated in the manuscript.

We thank the reviewer for this thoughtful comment. We agree that surgically induced osteochondral defects (OCDs) differ from the changes observed in naturally occurring TMJ-OA. However, OCDs have been well established in the literature as reproducible injury models for the development and evaluation of regenerative therapies for OA. On this basis, we believe the framing of our study is appropriate, as our aim was to establish and characterize a reproducible defect model rather than to replicate the full pathophysiology of naturally occurring TMJ-OA.

To address the reviewer's concern and avoid overstating our findings, we have revised the title and manuscript text to clarify that our model produces osteoarthritis-like changes and is intended as a platform for future regenerative studies, rather than as a direct model of natural TMJ-OA. The revised title now reads: "Goat temporomandibular joint osteochondral defects for future regenerative studies of osteoarthritis-like degeneration".

Regarding the application of this model for regenerative therapies, we acknowledge that this was not directly demonstrated in the manuscript. We have revised the relevant statements to present this as a potential future application rather than a validated outcome of the current study and have tempered the language accordingly in the Abstract, Introduction, Results, Discussion, and Conclusion.

We appreciate the reviewer's attention to these important points, which have helped us improve the clarity and accuracy of the manuscript. We hope these revisions address the reviewer's concerns.

2. **There is no mechanistic evidence showing that the current model recapitulates the progression of natural TMJ osteoarthritis. The authors should acknowledge this limitation and adopt a more cautious interpretation regarding the model's relevance for future regenerative therapy studies.**

We thank the reviewer for this important observation. We agree that our current model does not provide mechanistic evidence demonstrating that it recapitulates the progression of natural TMJ osteoarthritis. This has now been explicitly acknowledged as a limitation in the revised manuscript (see Discussion). Specifically, we have added the following clarification: "While the OCDs described here induce cartilage and subchondral bone changes, they do not necessarily

reproduce the gradual, multifactorial progression characteristic of naturally occurring TMJ-OA. Natural TMJ-OA develops through a complex, multifactorial process involving mechanical, biochemical, and inflammatory pathways that unfold gradually over time, whereas the OCDs described here produce a localized injury with a distinct and likely faster progression. As such, this model should currently be regarded as a reproducible platform for evaluating the efficacy of regenerative therapies on a defined OCD, rather than a validated surrogate for natural TMJ-OA pathogenesis. As such, findings from this model should be interpreted with caution when extrapolating to the pathophysiology of natural disease progression".

3. Macroscopic analysis: In the current representative images, macroscopic changes are difficult to appreciate. Higher-magnification images should be provided to demonstrate features such as surface roughness, irregularity, fibrillation, erosion, and ulceration. The images should include clear arrow annotations and scale bars to support the reported OARSI scores.

We thank the reviewer for this comment. We would like to clarify that the macroscopic images already include a ruler positioned beneath the condyle, which serves as a scale reference, allowing the reader to appreciate the actual size and extent of the surface lesions relative to the joint structure.

Regarding higher-magnification images, we chose to present the condyle at a magnification that preserves the full anatomical context of the joint surface, as we believe this is important for the reader to accurately localize the lesions relative to anatomical landmarks. Cropping to a higher magnification would improve the visibility of fine surface texture but at the cost of the broader anatomical context, which we consider equally important for interpreting the OARSI macroscopic scoring, as this system evaluates lesion location and extent across the joint surface rather than surface texture alone. That said, we agree that highlighting the specific features underlying the reported scores would improve clarity. We have therefore added a new supplementary figure to indicate OA-like changes (**see S6 Figure**). We hope this addresses the reviewer's concern while preserving the anatomical context, which we believe is important for interpreting the macroscopic findings.

4. Microscopic analysis: It appears that the N values in the microscopic analysis represent the number of sections rather than the number of animals. It is strongly recommended to present animal-level data (i.e., averaging all sections from each animal and plotting group comparisons such as n=3 vs n=4 vs n=6). While an additional analysis using an identical number of sections per animal could be included, this approach is less preferable due to potential selection bias.

We thank the reviewer for this important observation. In the revised manuscript, we have re-analyzed the microscopic data using the animal as the unit of analysis: for each animal, scores were averaged across all examined sections, and these animal-level means were then used for group comparisons (n = 3/goat/OCD type). This animal-based analysis is now presented in **the revised Figure 9** and corresponding text. We would like to clarify that, given the size of the condyle, it was not feasible to section and exhaustively analyze the entire structure. Sections were therefore selected in which the OCD was clearly visible, ensuring that the analyzed

sections accurately captured the region of interest in each animal. This selection criterion was applied consistently and objectively across all animals and groups, based on defect visibility rather than any outcome-related feature, and therefore does not introduce the type of selection bias that the reviewer raised. We appreciate the reviewer's suggestion to additionally consider an analysis using an identical number of sections per animal. However, because section selection was already based on defect visibility rather than an arbitrary fixed number, we believe our approach minimizes selection bias while remaining practically feasible given the anatomical constraints of the condyle. For this reason, we have retained the animal-averaged approach based on defect-visible sections as our primary analysis.

5. Apparently, N numbers in the microscopic analysis represent the number of sections examined rather than the number of animals. It is strongly recommended to show animal-based plot (i.e., average of entire sections from each animal in each group; plot of 3 vs 4 vs 6). In a separate plot, they may include an identical number of sections (e.g., two sections from each goat) to compare. This latter approach is not encouraged due to the selection bias.

We thank the reviewer for this important methodological observation. In the revised manuscript, we have re-analyzed the microscopic data using the animal as the unit of analysis, calculating the average score across 2-3 examined sections for each individual animal, and then comparing these animal-level averages with controls.

We have noted this rationale explicitly in the revised Methods and Results section.

6. The current dataset provides an opportunity to estimate the statistical power and inform sample size calculations for future studies. For example, the authors could estimate the number of animals required to achieve 80% power ($\alpha = 0.05$) to detect a 50% reduction in OARSI scores with a therapeutic intervention.

We thank the reviewer for this valuable suggestion. Considering histological scoring is the gold-standard OARSI outcome for cartilage repair/degeneration studies, using the Safranin O OARSI score data from the current study, we performed post hoc power analyses to estimate the sample size required to detect a 50% reduction in OARSI score with a therapeutic intervention, assuming $\alpha = 0.05$ and 80% power. Sample sizes were initially calculated for a two-sample t-test and inflated by a factor of 1.15 to account for the reduced efficiency of the Mann–Whitney U test, appropriate for the ordinal OARSI score data. Based on this analysis, a sample size of 7 animals per group would be required for a study using the Superior Defect (mean ± SD = 5.11 ± 1.35; Cohen's d = 1.89), or 6 animals per group for the Tunnel Defect (mean ± SD = 4.52 ± 0.97; Cohen's d = 2.33).

We have added this information to the Discussion section to guide sample size planning for future regenerative therapy studies using this model.

7. The use of unoperated animals as controls is suboptimal. A more appropriate control would be sham-operated animals (i.e., anesthesia, incision, and surgical exposure without drilling). The potential impact of this limitation should be explicitly discussed.

We thank the reviewer for this important observation. We agree that sham-operated controls (anesthesia, incision, and surgical exposure without drilling) would represent a more rigorous control condition, as they would help isolate the specific effects of the OCD from those attributable to surgical exposure, anesthesia, and associated inflammatory responses alone. We acknowledge this as a limitation of the current study design and have added the following discussion point to the revised manuscript:

A limitation of this study is the use of unoperated controls rather than sham-operated controls. While unoperated controls allowed us to characterize baseline TMJ morphology and histology, they do not account for potential confounding effects of anesthesia, surgical exposure, and incision-related inflammation that may independently influence the joint tissue response. Future studies incorporating sham-operated controls would allow more precise attribution of the observed changes specifically to the osteochondral defect itself, rather than to the surgical procedure more broadly. We would also like to highlight that, despite this limitation, the unoperated control joints in this study provided valuable baseline histological data. Detailed histological characterization of the aged goat TMJ is currently limited in the literature, and we believe the baseline histological data presented here help address this gap by providing a normative reference for TMJ condylar cartilage and subchondral bone architecture in this large-animal model. We have noted this contribution explicitly in the revised Discussion.

Sincerely,

Dr. Alejandro J. Almarza
Professor
Director, Collaborative for REsearch to Advance TMD Evidence (CREATE) Center
Department of Oral and Craniofacial Sciences
University of Pittsburgh
