# Supplementary material for: Goat temporomandibular joint osteochondral defects for future regenerative studies of osteoarthritis-like degeneration
Source: PLoS One. 2026 Aug 19;21(8):e0356421. doi: 10.1371/journal.pone.0356421 (PMC13489421; doi:10.1371/journal.pone.0356421)
Supplement: Supplementary file 4 [file pone.0356421.s011.docx]

**Title:**

Goat temporomandibular joint osteochondral defects for future regenerative studies of osteoarthritis-like degeneration

**Authors:**

Mairobys Socorro, Xudong Dong, Juan M Taboas, Alejandro J Almarza

We sincerely thank the reviewer for the valuable feedback, which has substantially improved the manuscript.

**Point-by-point response to reviewer #1:**

1. **I thank the authors for addressing all concerns. I have one more minor suggestion to improve the presentation. Supplemental Figure 1 shows nice illustrations for both models, and each panel of sFig 1 can be incorporated into Figure 1 and Figure 2. This should improve the clarity of the model for readers outside the field.**

We have revised the manuscript accordingly. Specifically, we updated the legends of Fig 1 and Fig 2, removed S1 Fig, and renumbered the remaining supplementary figures accordingly. We also improved the manuscript's grammar.

**2. The data should be provided as part of the manuscript or its supporting information, or deposited to a public repository. For example, in addition to summary statistics, the data points behind means, medians and variance measures should be available.**

All data are in the manuscript and/or supporting information files. The data points behind means and SDs have been included in the manuscript as supporting information (Excel file).
