# Supplementary material for: Goat temporomandibular joint osteochondral defects for future regenerative studies of osteoarthritis-like degeneration
Source: PLoS One. 2026 Aug 19;21(8):e0356421. doi: 10.1371/journal.pone.0356421 (PMC13489421; doi:10.1371/journal.pone.0356421)
Supplement: Peer review history

# Peer review: Decision Letter 0

**DOI:** 10.1371/journal.pone.0356421.r001

**Authors:** James Cray

26 Jun 2026

Dear Dr. Almarza,

Thank you for submitting your manuscript to PLOS ONE. After careful consideration, we feel that it has merit but does not fully meet PLOS ONE’s publication criteria as it currently stands. Therefore, we invite you to submit a revised version of the manuscript that addresses the points raised during the review process.

Major revision is warranted given clarity, the discussion which should be reworked to better reflect the results, and a question about representative images.

Please submit your revised manuscript by Aug 25 2026 11:59PM. If you will need more time than this to complete your revisions, please reply to this message or contact the journal office at plosone@plos.org. When you're ready to submit your revision, log on to https://www.editorialmanager.com/pone/ and select the 'Submissions Needing Revision' folder to locate your manuscript file.

- A letter that responds to each point raised by the academic editor and reviewer(s). You should upload this letter as a separate file labeled 'Response to Reviewers'.
- A marked-up copy of your manuscript that highlights changes made to the original version. You should upload this as a separate file labeled 'Revised Manuscript with Track Changes'.
- An unmarked version of your revised paper without tracked changes. You should upload this as a separate file labeled 'Manuscript'.

If you would like to make changes to your financial disclosure, please include your updated statement in your cover letter. Guidelines for resubmitting your figure files are available below the reviewer comments at the end of this letter.

If applicable, we recommend that you deposit your laboratory protocols in protocols.io to enhance the reproducibility of your results. Protocols.io assigns your protocol its own identifier (DOI) so that it can be cited independently in the future. For instructions see: https://journals.plos.org/plosone/s/submission-guidelines#loc-laboratory-protocols. Additionally, PLOS ONE offers an option for publishing peer-reviewed Lab Protocol articles, which describe protocols hosted on protocols.io. Read more information on sharing protocols at https://plos.org/protocols?utm_medium=editorial-email&utm_source=authorletters&utm_campaign=protocols.

As the corresponding author, your ORCID iD is verified in the submission system and will appear in the published article. PLOS supports the use of ORCID, and we encourage all coauthors to register for an ORCID iD and use it as well. Please encourage your coauthors to verify their ORCID iD within the submission system before final acceptance, as unverified ORCID iDs will not appear in the published article. *Only*  the individual author can complete the verification step; PLOS staff *cannot*  verify ORCID iDs on behalf of authors.

We look forward to receiving your revised manuscript.

Kind regards,

James J Cray Jr., Ph.D.

Academic Editor

PLOS One

**Journal Requirements:**

When submitting your revision, we need you to address these additional requirements.

1. Please ensure that your manuscript meets PLOS ONE's style requirements, including those for file naming. The PLOS ONE style templates can be found at

https://journals.plos.org/plosone/s/file?id=wjVg/PLOSOne_formatting_sample_main_body.pdf and

https://journals.plos.org/plosone/s/file?id=ba62/PLOSOne_formatting_sample_title_authors_affiliations.pdf

2. We note that the grant information you provided in the ‘Funding Information’ and ‘Financial Disclosure’ sections do not match.

When you resubmit, please ensure that you provide the correct grant numbers for the awards you received for your study in the ‘Funding Information’ section.

3. Please note that funding information should not appear in any section or other areas of your manuscript. We will only publish funding information present in the Funding Statement section of the online submission form. Please remove any funding-related text from the manuscript.

4. When completing the data availability statement of the submission form, you indicated that you will make your data available on acceptance. We strongly recommend all authors decide on a data sharing plan before acceptance, as the process can be lengthy and hold up publication timelines. Please note that, though access restrictions are acceptable now, your entire data will need to be made freely accessible if your manuscript is accepted for publication. This policy applies to all data except where public deposition would breach compliance with the protocol approved by your research ethics board. If you are unable to adhere to our open data policy, please kindly revise your statement to explain your reasoning and we will seek the editor's input on an exemption. Please be assured that, once you have provided your new statement, the assessment of your exemption will not hold up the peer review process.

5. We note that Figure 1, 2, and 8; Supporting Information Figure 2 in your submission contain copyrighted images. All PLOS content is published under the Creative Commons Attribution License (CC BY 4.0), which means that the manuscript, images, and Supporting Information files will be freely available online, and any third party is permitted to access, download, copy, distribute, and use these materials in any way, even commercially, with proper attribution. For more information, see our copyright guidelines: http://journals.plos.org/plosone/s/licenses-and-copyright.

We require you to either (1) present written permission from the copyright holder to publish these figures specifically under the CC BY 4.0 license, or (2) remove the figures from your submission:

1. You may seek permission from the original copyright holder of Figures 1, 2, and 8; Supporting Information Figure 2  to publish the content specifically under the CC BY 4.0 license.

We recommend that you contact the original copyright holder with the Content Permission Form (http://journals.plos.org/plosone/s/file?id=7c09/content-permission-form.pdf) and the following text:

“I request permission for the open-access journal PLOS ONE to publish XXX under the Creative Commons Attribution License (CCAL) CC BY 4.0 (http://creativecommons.org/licenses/by/4.0/). Please be aware that this license allows unrestricted use and distribution, even commercially, by third parties. Please reply and provide explicit written permission to publish XXX under a CC BY license and complete the attached form.”

Please upload the completed Content Permission Form or other proof of granted permissions as an "Other" file with your submission.

In the figure caption of the copyrighted figure, please include the following text: “Reprinted from [ref] under a CC BY license, with permission from [name of publisher], original copyright [original copyright year].”

2. If you are unable to obtain permission from the original copyright holder to publish these figures under the CC BY 4.0 license or if the copyright holder’s requirements are incompatible with the CC BY 4.0 license, please either i) remove the figure or ii) supply a replacement figure that complies with the CC BY 4.0 license. Please check copyright information on all replacement figures and update the figure caption with source information. If applicable, please specify in the figure caption text when a figure is similar but not identical to the original image and is therefore for illustrative purposes only.

6. If the reviewer comments include a recommendation to cite specific previously published works, please review and evaluate these publications to determine whether they are relevant and should be cited. There is no requirement to cite these works unless the editor has indicated otherwise.

[Note: HTML markup is below. Please do not edit.]

Reviewers' comments:

Reviewer's Responses to Questions

**Comments to the Author**

1. Is the manuscript technically sound, and do the data support the conclusions?

Reviewer #1: Partly

**********

2. Has the statistical analysis been performed appropriately and rigorously?

Reviewer #1: No

**********

3. Have the authors made all data underlying the findings in their manuscript fully available?

The PLOS Data policy

Reviewer #1: Yes

**********

4. Is the manuscript presented in an intelligible fashion and written in standard English?

Reviewer #1: Yes

**********

Reviewer #1: This manuscript describes a goat model of temporomandibular joint (TMJ) osteochondral defects, comparing a novel tunnel defect with a conventional superior defect. The study addresses an important gap in preclinical TMJ models and provides potentially useful methodology for regenerative applications. The use of skeletally mature female goats and the comparison of two surgical approaches are notable strengths. Although the sample sizes are relatively small, this study can serve as an important pilot for future mechanistic and translational investigations. I have several suggestions to strengthen the quantification and presentation of the data, as outlined below.

1. The title can be misleading and needs to be revised. The current title is potentially misleading and should be revised. Cartilage damage-induced changes in adjacent tissue are likely distinct from those observed in natural TMJ osteoarthritis. Furthermore, the application of this model for regenerative therapies is not directly demonstrated in the manuscript.

2. There is no mechanistic evidence showing that the current model recapitulates the progression of natural TMJ osteoarthritis. The authors should acknowledge this limitation and adopt a more cautious interpretation regarding the model’s relevance for future regenerative therapy studies.

3. Macroscopic analysis: In the current representative images, macroscopic changes are difficult to appreciate. Higher-magnification images should be provided to demonstrate features such as surface roughness, irregularity, fibrillation, erosion, and ulceration. The images should include clear arrow annotations and scale bars to support the reported OARSI scores.

4. Microscopic analysis: It appears that the N values in the microscopic analysis represent the number of sections rather than the number of animals. It is strongly recommended to present animal-level data (i.e., averaging all sections from each animal and plotting group comparisons such as n=3 vs n=4 vs n=6). While an additional analysis using an identical number of sections per animal could be included, this approach is less preferable due to potential selection bias.

5. Apparently, N numbers in the microscopic analysis represent the number of sections examined rather than the number of animals. It is strongly recommended to show animal-based plot (i.e., average of entire sections from each animal in each group; plot of 3 vs 4 vs 6). In a separate plot, they may include an identical number of sections (e.g., two sections from each goat) to compare. This latter approach is not encouraged due to the selection bias.

6. The current dataset provides an opportunity to estimate the statistical power and inform sample size calculations for future studies. For example, the authors could estimate the number of animals required to achieve 80% power (α = 0.05) to detect a 50% reduction in OARSI scores with a therapeutic intervention.

7. The use of unoperated animals as controls is suboptimal. A more appropriate control would be sham-operated animals (i.e., anesthesia, incision, and surgical exposure without drilling). The potential impact of this limitation should be explicitly discussed.

**********

what does this mean?). If published, this will include your full peer review and any attached files.

If you choose “no”, your identity will remain anonymous but your review may still be made public.

**Do you want your identity to be public for this peer review?**  For information about this choice, including consent withdrawal, please see our Privacy Policy

Reviewer #1: **Yes:**  Man-Kyo Chung

**********

[NOTE: If reviewer comments were submitted as an attachment file, they will be attached to this email and accessible via the submission site. Please log into your account, locate the manuscript record, and check for the action link "View Attachments". If this link does not appear, there are no attachment files.]

To ensure your figures meet our technical requirements, please review our figure guidelines: https://journals.plos.org/plosone/s/figures

You may also use PLOS’s free figure tool, NAAS, to help you prepare publication quality figures: https://journals.plos.org/plosone/s/figures#loc-tools-for-figure-preparation.

NAAS will assess whether your figures meet our technical requirements by comparing each figure against our figure specifications.

# Author comment: Author response to Decision Letter 1

**DOI:** 10.1371/journal.pone.0356421.r002

20 Jul 2026

We sincerely thank the editor and reviewer for the insightful comments and constructive feedback. We have carefully addressed each comment and revised the manuscript accordingly. We believe that the reviewers’ suggestions have significantly strengthened our manuscript. The discussion has been reworked to better reflect the results.

Point-to-point response to the Academic Editor:

1. Please ensure that your manuscript meets PLOS ONE's style requirements, including those for file naming. The manuscript has been revised to comply with PLOS ONE's style requirements.

2. We note that the grant information you provided in the ‘Funding Information’ and ‘Financial Disclosure’ sections do not match. We have provided the correct grant numbers for this study in the ‘Funding Information’ section.

3. Please note that funding information should not appear in any section or other areas of your manuscript. We removed all funding-related text from the manuscript.

4. When completing the data availability statement of the submission form, you indicated that you will make your data available on acceptance. We have clarified that “All data are in the manuscript and/or supporting information files”

5. We note that Figure 1, 2, and 8; Supporting Information Figure 2 in your submission contain copyrighted images. Thank you for your careful review of our submission and for bringing this matter to our attention. We would like to respectfully clarify that Figures 1, 2, and 8, as well as Supporting Information Figure 2, are entirely original and were produced by the authors. These figures do not contain any copyrighted or third-party material, and no images were reproduced or adapted from previously published sources.

6. If the reviewer comments include a recommendation to cite specific previously published works, please review and evaluate these publications to determine whether they are relevant and should be cited. N/A

Point-by-point response to reviewer #1:

1. The title can be misleading and needs to be revised. The current title is potentially misleading and should be revised. Cartilage damage-induced changes in adjacent tissue are likely distinct from those observed in natural TMJ osteoarthritis. Furthermore, the application of this model for regenerative therapies is not directly demonstrated in the manuscript.

We thank the reviewer for this thoughtful comment. We agree that surgically induced osteochondral defects (OCDs) differ from the changes observed in naturally occurring TMJ-OA. However, OCDs have been well established in the literature as reproducible injury models for the development and evaluation of regenerative therapies for OA. On this basis, we believe the framing of our study is appropriate, as our aim was to establish and characterize a reproducible defect model rather than to replicate the full pathophysiology of naturally occurring TMJ-OA.

To address the reviewer's concern and avoid overstating our findings, we have revised the title and manuscript text to clarify that our model produces osteoarthritis-like changes and is intended as a platform for future regenerative studies, rather than as a direct model of natural TMJ-OA. The revised title now reads: “Goat temporomandibular joint osteochondral defects for future regenerative studies of osteoarthritis-like degeneration”.

Regarding the application of this model for regenerative therapies, we acknowledge that this was not directly demonstrated in the manuscript. We have revised the relevant statements to present this as a potential future application rather than a validated outcome of the current study and have tempered the language accordingly in the Abstract, Introduction, Results, Discussion, and Conclusion.

We appreciate the reviewer's attention to these important points, which have helped us improve the clarity and accuracy of the manuscript. We hope these revisions address the reviewer's concerns.

2. There is no mechanistic evidence showing that the current model recapitulates the progression of natural TMJ osteoarthritis. The authors should acknowledge this limitation and adopt a more cautious interpretation regarding the model’s relevance for future regenerative therapy studies.

We thank the reviewer for this important observation. We agree that our current model does not provide mechanistic evidence demonstrating that it recapitulates the progression of natural TMJ osteoarthritis. This has now been explicitly acknowledged as a limitation in the revised manuscript (see Discussion). Specifically, we have added the following clarification: "While the OCDs described here induce cartilage and subchondral bone changes, they do not necessarily reproduce the gradual, multifactorial progression characteristic of naturally occurring TMJ-OA. Natural TMJ-OA develops through a complex, multifactorial process involving mechanical, biochemical, and inflammatory pathways that unfold gradually over time, whereas the OCDs described here produce a localized injury with a distinct and likely faster progression. As such, this model should currently be regarded as a reproducible platform for evaluating the efficacy of regenerative therapies on a defined OCD, rather than a validated surrogate for natural TMJ-OA pathogenesis. As such, findings from this model should be interpreted with caution when extrapolating to the pathophysiology of natural disease progression”.

3. Macroscopic analysis: In the current representative images, macroscopic changes are difficult to appreciate. Higher-magnification images should be provided to demonstrate features such as surface roughness, irregularity, fibrillation, erosion, and ulceration. The images should include clear arrow annotations and scale bars to support the reported OARSI scores.

We thank the reviewer for this comment. We would like to clarify that the macroscopic images already include a ruler positioned beneath the condyle, which serves as a scale reference, allowing the reader to appreciate the actual size and extent of the surface lesions relative to the joint structure. Regarding higher-magnification images, we chose to present the condyle at a magnification that preserves the full anatomical context of the joint surface, as we believe this is important for the reader to accurately localize the lesions relative to anatomical landmarks. Cropping to a higher magnification would improve the visibility of fine surface texture but at the cost of the broader anatomical context, which we consider equally important for interpreting the OARSI macroscopic scoring, as this system evaluates lesion location and extent across the joint surface rather than surface texture alone. That said, we agree that highlighting the specific features underlying the reported scores would improve clarity. We have therefore added a new supplementary figure to indicate OA-like changes (see S6 Fig). We hope this addresses the reviewer's concern while preserving the anatomical context, which we believe is important for interpreting the macroscopic findings.

4. Microscopic analysis: It appears that the N values in the microscopic analysis represent the number of sections rather than the number of animals. It is strongly recommended to present animal-level data (i.e., averaging all sections from each animal and plotting group comparisons such as n=3 vs n=4 vs n=6). While an additional analysis using an identical number of sections per animal could be included, this approach is less preferable due to potential selection bias.

We thank the reviewer for this important observation. In the revised manuscript, we have re-analyzed the microscopic data using the animal as the unit of analysis: for each animal, scores were averaged across all examined sections, and these animal-level means were then used for group comparisons (n = 3/goat/OCD type). This animal-based analysis is now presented in the revised Figure 9 and corresponding text. We would like to clarify that, given the size of the condyle, it was not feasible to section and exhaustively analyze the entire structure. Sections were therefore selected in which the OCD was clearly visible, ensuring that the analyzed sections accurately captured the region of interest in each animal. This selection criterion was applied consistently and objectively across all animals and groups, based on defect visibility rather than any outcome-related feature, and therefore does not introduce the type of selection bias that the reviewer raised. We appreciate the reviewer's suggestion to additionally consider an analysis using an identical number of sections per animal. However, because section selection was already based on defect visibility rather than an arbitrary fixed number, we believe our approach minimizes selection bias while remaining practically feasible given the anatomical constraints of the condyle. For this reason, we have retained the animal-averaged approach based on defect-visible sections as our primary analysis.

5. Apparently, N numbers in the microscopic analysis represent the number of sections examined rather than the number of animals. It is strongly recommended to show animal based plot (i.e., average of entire sections from each animal in each group; plot of 3 vs 4 vs 6). In a separate plot, they may include an identical number of sections (e.g., two sections from each goat) to compare. This latter approach is not encouraged due to the selection bias.

We thank the reviewer for this important methodological observation. In the revised manuscript, we have re-analyzed the microscopic data using the animal as the unit of analysis, calculating the average score across 2-3 examined sections for each individual animal, and then comparing these animal-level averages with controls. We have noted this rationale explicitly in the revised Methods and Results section.

6. The current dataset provides an opportunity to estimate the statistical power and inform sample size calculations for future studies. For example, the authors could estimate the number of animals required to achieve 80% power (α = 0.05) to detect a 50% reduction in OARSI scores with a therapeutic intervention.

We thank the reviewer for this valuable suggestion. Considering histological scoring is the gold standard OARSI outcome for cartilage repair/degeneration studies, using the Safranin O OARSI score data from the current study, we performed post hoc power analyses to estimate the sample size required to detect a 50% reduction in OARSI score with a therapeutic intervention, assuming α = 0.05 and 80% power. Sample sizes were initially calculated for a two-sample t test and inflated by a factor of 1.15 to account for the reduced efficiency of the Mann–Whitney U test, appropriate for the ordinal OARSI score data. Based on this analysis, a sample size of 7 animals per group would be required for a study using the Superior Defect (mean ± SD = 5.11 ± 1.35; Cohen's d = 1.89), or 6 animals per group for the Tunnel Defect (mean ± SD = 4.52 ± 0.97; Cohen's d = 2.33).

We have added this information to the Discussion section to guide sample size planning for future regenerative therapy studies using this model.

7. The use of unoperated animals as controls is suboptimal. A more appropriate control would be sham-operated animals (i.e., anesthesia, incision, and surgical exposure without drilling). The potential impact of this limitation should be explicitly discussed.

We thank the reviewer for this important observation. We agree that sham-operated controls (anesthesia, incision, and surgical exposure without drilling) would represent a more rigorous control condition, as they would help isolate the specific effects of the OCD from those attributable to surgical exposure, anesthesia, and associated inflammatory responses alone. We acknowledge this as a limitation of the current study design and have added the following discussion point to the revised manuscript:

A limitation of this study is the use of unoperated controls rather than sham-operated controls. While unoperated controls allowed us to characterize baseline TMJ morphology and histology, they do not account for potential confounding effects of anesthesia, surgical exposure, and incision-related inflammation that may independently influence the joint tissue response. Future studies incorporating sham-operated controls would allow more precise attribution of the observed changes specifically to the osteochondral defect itself, rather than to the surgical procedure more broadly. We would also like to highlight that, despite this limitation, the unoperated control joints in this study provided valuable baseline histological data. Detailed histological characterization of the aged goat TMJ is currently limited in the literature, and we believe the baseline histological data presented here help address this gap by providing a normative reference for TMJ condylar cartilage and subchondral bone architecture in this large animal model. We have noted this contribution explicitly in the revised Discussion.

Attachment Submitted filename: Response to Reviewers.pdf

# Peer review: Decision Letter 1

**DOI:** 10.1371/journal.pone.0356421.r003

**Authors:** James Cray

30 Jul 2026

Dear Dr. Almarza,

Thank you for submitting your manuscript to PLOS ONE. After careful consideration, we feel that it has merit but does not fully meet PLOS ONE’s publication criteria as it currently stands. Therefore, we invite you to submit a revised version of the manuscript that addresses the points raised during the review process.

Please submit your revised manuscript by Aug 29 2026 11:59PM. If you will need more time than this to complete your revisions, please reply to this message or contact the journal office at plosone@plos.org. When you're ready to submit your revision, log on to https://www.editorialmanager.com/pone/ and select the 'Submissions Needing Revision' folder to locate your manuscript file.

- A letter that responds to each point raised by the academic editor and reviewer(s). You should upload this letter as a separate file labeled 'Response to Reviewers'.
- A marked-up copy of your manuscript that highlights changes made to the original version. You should upload this as a separate file labeled 'Revised Manuscript with Track Changes'.
- An unmarked version of your revised paper without tracked changes. You should upload this as a separate file labeled 'Manuscript'.

If you would like to make changes to your financial disclosure, please include your updated statement in your cover letter. Guidelines for resubmitting your figure files are available below the reviewer comments at the end of this letter.

If applicable, we recommend that you deposit your laboratory protocols in protocols.io to enhance the reproducibility of your results. Protocols.io assigns your protocol its own identifier (DOI) so that it can be cited independently in the future. For instructions see: https://journals.plos.org/plosone/s/submission-guidelines#loc-laboratory-protocols. Additionally, PLOS ONE offers an option for publishing peer-reviewed Lab Protocol articles, which describe protocols hosted on protocols.io. Read more information on sharing protocols at https://plos.org/protocols?utm_medium=editorial-email&utm_source=authorletters&utm_campaign=protocols.

As the corresponding author, your ORCID iD is verified in the submission system and will appear in the published article. PLOS supports the use of ORCID, and we encourage all coauthors to register for an ORCID iD and use it as well. Please encourage your coauthors to verify their ORCID iD within the submission system before final acceptance, as unverified ORCID iDs will not appear in the published article. *Only*  the individual author can complete the verification step; PLOS staff *cannot*  verify ORCID iDs on behalf of authors.

We look forward to receiving your revised manuscript.

Kind regards,

James J Cray Jr., Ph.D.

Academic Editor

PLOS One

Journal Requirements:

If the reviewer comments include a recommendation to cite specific previously published works, please review and evaluate these publications to determine whether they are relevant and should be cited. There is no requirement to cite these works unless the editor has indicated otherwise.

Please review your reference list to ensure that it is complete and correct. If you have cited papers that have been retracted, please include the rationale for doing so in the manuscript text, or remove these references and replace them with relevant current references. Any changes to the reference list should be mentioned in the rebuttal letter that accompanies your revised manuscript. If you need to cite a retracted article, indicate the article’s retracted status in the References list and also include a citation and full reference for the retraction notice.

[Note: HTML markup is below. Please do not edit.]

Reviewers' comments:

Reviewer's Responses to Questions

**Comments to the Author**

Reviewer #1: All comments have been addressed

**********

2. Is the manuscript technically sound, and do the data support the conclusions?

Reviewer #1: Yes

**********

3. Has the statistical analysis been performed appropriately and rigorously?

Reviewer #1: Yes

**********

4. Have the authors made all data underlying the findings in their manuscript fully available?

The PLOS Data policy

Reviewer #1: No

**********

5. Is the manuscript presented in an intelligible fashion and written in standard English?

Reviewer #1: Yes

**********

Reviewer #1: I thank the authors for addressing all concerns. I have one more minor suggestion to improve the presentation. Supplemental Figure 1 shows nice illustrations for both models, and each panel of sFig 1 can be incorporated into Figure 1 and Figure 2. This should improve the clarity of the model for readers outside the field.

**********

what does this mean?). If published, this will include your full peer review and any attached files.

If you choose “no”, your identity will remain anonymous but your review may still be made public.

**Do you want your identity to be public for this peer review?**  For information about this choice, including consent withdrawal, please see our Privacy Policy

Reviewer #1: No

**********

[NOTE: If reviewer comments were submitted as an attachment file, they will be attached to this email and accessible via the submission site. Please log into your account, locate the manuscript record, and check for the action link "View Attachments". If this link does not appear, there are no attachment files.]

To ensure your figures meet our technical requirements, please review our figure guidelines: https://journals.plos.org/plosone/s/figures

You may also use PLOS’s free figure tool, NAAS, to help you prepare publication quality figures: https://journals.plos.org/plosone/s/figures#loc-tools-for-figure-preparation.

NAAS will assess whether your figures meet our technical requirements by comparing each figure against our figure specifications.

# Author comment: Author response to Decision Letter 2

**DOI:** 10.1371/journal.pone.0356421.r004

31 Jul 2026

Point-by-point response to reviewer #1:

1. I thank the authors for addressing all concerns. I have one more minor suggestion to improve the presentation. Supplemental Figure 1 shows nice illustrations for both models, and each panel of sFig 1 can be incorporated into Figure 1 and Figure 2. This should improve the clarity of the model for readers outside the field.

We have revised the manuscript accordingly. Specifically, we updated the legends of Fig 1 and Fig 2, removed S1 Fig, and renumbered the remaining supplementary figures accordingly. We also improved the manuscript's grammar.

2. The data should be provided as part of the manuscript or its supporting information, or deposited to a public repository. For example, in addition to summary statistics, the data points behind means, medians and variance measures should be available.

All data are in the manuscript and/or supporting information files. The data points behind means and SDs have been included in the manuscript as supporting information (Excel file).

Attachment Submitted filename: Response to Reviewers.docx

# Editor report: Decision Letter 2

**DOI:** 10.1371/journal.pone.0356421.r005

**Authors:** James Cray

3 Aug 2026

Goat temporomandibular joint osteochondral defects for future regenerative studies of osteoarthritis-like degeneration

PONE-D-26-26485R2

Dear Dr. Almarza,

We’re pleased to inform you that your manuscript has been judged scientifically suitable for publication and will be formally accepted for publication once it meets all outstanding technical requirements.

Within one week, you’ll receive an e-mail detailing the required amendments. When these have been addressed, you’ll receive a formal acceptance letter and your manuscript will be scheduled for publication.

An invoice will be generated when your article is formally accepted. Please note, if your institution has a publishing partnership with PLOS and your article meets the relevant criteria, all or part of your publication costs will be covered. Please make sure your user information is up-to-date by logging into Editorial Manager at Editorial Manager® and clicking the ‘Update My Information' link at the top of the page. For questions related to billing, please contact billing support.

If your institution or institutions have a press office, please notify them about your upcoming paper to help maximize its impact. If they’ll be preparing press materials, please inform our press team as soon as possible -- no later than 48 hours after receiving the formal acceptance. Your manuscript will remain under strict press embargo until 2 pm Eastern Time on the date of publication. For more information, please contact onepress@plos.org.

Kind regards,

James J Cray Jr., Ph.D.

Academic Editor

PLOS One

Additional Editor Comments (optional):

Reviewers' comments:

# Editor report: Acceptance letter

**DOI:** 10.1371/journal.pone.0356421.r006

**Authors:** James Cray

PONE-D-26-26485R2

PLOS One

Dear Dr. Almarza,

I'm pleased to inform you that your manuscript has been deemed suitable for publication in PLOS One. Congratulations! Your manuscript is now being handed over to our production team.

At this stage, our production department will prepare your paper for publication. This includes ensuring the following:

* All references, tables, and figures are properly cited

* All relevant supporting information is included in the manuscript submission,

* There are no issues that prevent the paper from being properly typeset

You will receive further instructions from the production team, including instructions on how to review your proof when it is ready. Please keep in mind that we are working through a large volume of accepted articles, so please give us a few days to review your paper and let you know the next and final steps.

Lastly, if your institution or institutions have a press office, please let them know about your upcoming paper now to help maximize its impact. If they'll be preparing press materials, please inform our press team within the next 48 hours. Your manuscript will remain under strict press embargo until 2 pm Eastern Time on the date of publication. For more information, please contact onepress@plos.org.

You will receive an invoice from PLOS for your publication fee after your manuscript has reached the completed accept phase. If you receive an email requesting payment before acceptance or for any other service, this may be a phishing scheme. Learn how to identify phishing emails and protect your accounts at https://explore.plos.org/phishing.

If we can help with anything else, please email us at customercare@plos.org.

Thank you for submitting your work to PLOS One and supporting open access.

Kind regards,

PLOS ONE Editorial Office Staff

on behalf of

Dr. James J Cray Jr.

Academic Editor

PLOS One
